# Specialized interferon action in COVID-19

**DOI:** 10.1073/pnas.2116730119

**Published:** 2022-02-25

**Authors:** Matthew D. Galbraith, Kohl T. Kinning, Kelly D. Sullivan, Paula Araya, Keith P. Smith, Ross E. Granrath, Jessica R. Shaw, Ryan Baxter, Kimberly R. Jordan, Seth Russell, Monika Dzieciatkowska, Julie A. Reisz, Fabia Gamboni, Francesca Cendali, Tusharkanti Ghosh, Kejun Guo, Cara C. Wilson, Mario L. Santiago, Andrew A. Monte, Tellen D. Bennett, Kirk C. Hansen, Elena W. Y. Hsieh, Angelo D’Alessandro, Joaquin M. Espinosa

**Affiliations:** ^a^Linda Crnic Institute for Down Syndrome, University of Colorado Anschutz Medical Campus, Aurora, CO 80045;; ^b^Department of Pharmacology, University of Colorado Anschutz Medical Campus, Aurora, CO 80045;; ^c^Department of Pediatrics, Section of Developmental Biology, University of Colorado Anschutz Medical Campus, Aurora, CO 80045;; ^d^Department of Immunology and Microbiology, University of Colorado Anschutz Medical Campus, Aurora, CO 80045;; ^e^Data Science to Patient Value, University of Colorado Anschutz Medical Campus, Aurora, CO 80045;; ^f^Department of Biochemistry and Molecular Genetics, University of Colorado Anschutz Medical Campus, Aurora, CO 80045;; ^g^Department of Biostatistics and Informatics, Colorado School of Public Health, Aurora, CO 80045;; ^h^Department of Medicine, Division of Infectious Diseases, University of Colorado Anschutz Medical Campus, Aurora, CO 80045;; ^i^Department of Emergency Medicine, University of Colorado Anschutz Medical Campus, Aurora, CO 80045;; ^j^Department of Pediatrics, Sections of Informatics and Data Science and Critical Care Medicine, University of Colorado Anschutz Medical Campus, Aurora, CO 80045;; ^k^Department of Pediatrics, Section of Allergy/Immunology, University of Colorado Anschutz Medical Campus, Aurora, CO 80045

**Keywords:** COVID-19, interferon, SARS-CoV-2, CyTOF, cytokine

## Abstract

The impacts of interferon (IFN) signaling on COVID-19 pathology are multiple, with both protective and harmful effects being documented. We report here a multiomics investigation of systemic IFN signaling in hospitalized COVID-19 patients, defining the multiomics biosignatures associated with varying levels of 12 different type I, II, and III IFNs. The antiviral transcriptional response in circulating immune cells is strongly associated with a specific subset of IFNs, most prominently IFNA2 and IFNG. In contrast, proteomics signatures indicative of endothelial damage and platelet activation associate with high levels of IFNB1 and IFNA6. Seroconversion and time since hospitalization associate with a significant decrease in a specific subset of IFNs. Additionally, differential IFN subtype production is linked to distinct constellations of circulating myeloid and lymphoid immune cell types. Each IFN has a unique metabolic signature, with IFNG being the most associated with activation of the kynurenine pathway. IFNs also show differential relationships with clinical markers of poor prognosis and disease severity. For example, whereas IFNG has the strongest association with C-reactive protein and other immune markers of poor prognosis, IFNB1 associates with increased neutrophil to lymphocyte ratio, a marker of late severe disease. Altogether, these results reveal specialized IFN action in COVID-19, with potential diagnostic and therapeutic implications.

## Significance

The interferon (IFN) family of proteins plays key roles in the immune response against viruses and other pathogens. In the context of COVID-19, IFNs have been shown to be key for restraining SARS-CoV-2 infection but have also been described as drivers of severe symptoms. However, it is not fully understood how each member of the IFN family contributes to distinct aspects of COVID-19. We report here the results of a deep examination of 12 different IFNs in hospitalized COVID-19 patients, which revealed clear differences among IFNs in their associations with molecular, cellular, and physiological processes relevant to COVID-19 presentation.

The impact of interferon (IFN) signaling on the course of COVID-19 pathology has been the subject of much investigation, with both protective and deleterious effects being reported. There are three major types of IFN signaling defined by the transmembrane receptors and downstream signaling kinases engaged (1). Type I signaling involves IFN-α, -β, -ε, -κ, and -ω IFNs, the IFNAR1 and IFNAR2 receptors, and the downstream kinases JAK1 and TYK2. Type II signaling involves the IFNG, the IFNGR1 and IFNGR2 receptors, and the downstream kinases JAK1 and JAK2. Type III IFN signaling involves the λ IFNs, the IFNLR1 and IL10RB receptors, and the JAK1 and TYK2 kinases. However, this broad classification does not capture the biological complexity driven by subtypes acting through the same receptors. This is most evident by the differential effects of α-subtypes versus IFNB1 within type I signaling (1). Even within α-subtypes there is significant heterogeneity in cellular source, site of action, and downstream effects (1).

In the context of SARS-CoV-2 infections, the protective effects of IFN signaling are demonstrated by studies showing that severe COVID-19 is associated with decreased IFN signaling (2–4), the presence of autoantibodies blocking the action of specific IFNs (5–9), and genetic variants that impair IFN signaling (10, 11). In the nasal mucosa, autoantibodies targeting type I IFNs correlate with high viral load and severe COVID-19 (8). In the upper respiratory tract, high levels of type III IFNs, and to a lesser extent type I IFNs, are associated with reduced disease risk or severity (12). In bronchial aspirates, increased levels of type III IFNs correlate with lower viral load and faster clearance (3). However, type I IFN signaling has been established as a driver of pathology in mouse models of both SARS-CoV-1 and SARS-CoV-2 infections (13, 14), and type I and III IFNs have been implicated in disruption of lung barrier function and increased susceptibility to secondary bacterial infections in mice (15, 16). This duality in the role of IFN signaling could be explained in part by an untuned viral response during SARS-CoV-2 infections, whereby antiviral IFN signaling is delayed relative to proinflammatory signaling (3). Furthermore, the impacts of IFN signaling could vary at different sites, even along the upper versus lower respiratory tracts (12). This complexity has fueled the design of seemingly contradictory clinical trials using either IFNs (17) or agents that block IFN signaling, such as JAK inhibitors (18). Thus, it is possible that context-dependent variations in IFN signaling may attenuate or exacerbate COVID-19 pathology. Indeed, retrospective analysis of IFN-α2b treatment in COVID-19 showed that early administration was associated with reduced mortality, whereas late administration was associated with increased mortality (19).

Within this context, we report here a multiomics analysis of systemic IFN signaling in hospitalized COVID-19 patients, including a comprehensive examination of the whole-blood transcriptome, plasma proteome, anti–SARS-CoV-2 antibodies, peripheral immune cell repertoire, plasma and red blood cell (RBC) metabolomes, as well as immune and clinical markers of disease risk and severity in relationship to circulating levels of 12 different IFNs. These analyses revealed heterogeneity in the relationship between levels of each IFN and key molecular, cellular, metabolic, and physiological processes relevant to COVID-19 pathophysiology. These results indicate that modulation of IFN signaling in the clinic, with either agonists or antagonists, must take into account the endogenous state of the IFN milieu at the time of intervention, and that subtype-specific effects are to be expected.

## Results

### Variable IFN Signaling in COVID-19 Associates with Levels of a Specific Subset of IFNs.

We analyzed the datasets generated by the COVIDome Project (covidome.org) to investigate IFN signaling in hospitalized COVID-19 patients. The COVIDome Project datasets have been previously described (20, 21) and include matched whole-blood transcriptomes, plasma proteomics via complementary SOMAscan and mass spectrometry (MS) assays, measurement of 82 immune factors by multiplexed immunoassays, SARS-CoV-2 seroconversion assays, immune cell profiling by mass cytometry, plasma and RBC metabolomics, as well as annotated clinical metadata. The cohort analyzed in this study consists of 73 hospitalized COVID-19 patients with mild-to-moderate disease at the time of research blood draw and 32 controls (*SI Appendix*, *SI Extended Methods*; see Dataset S1 for cohort characteristics).

**Fig. 1.**
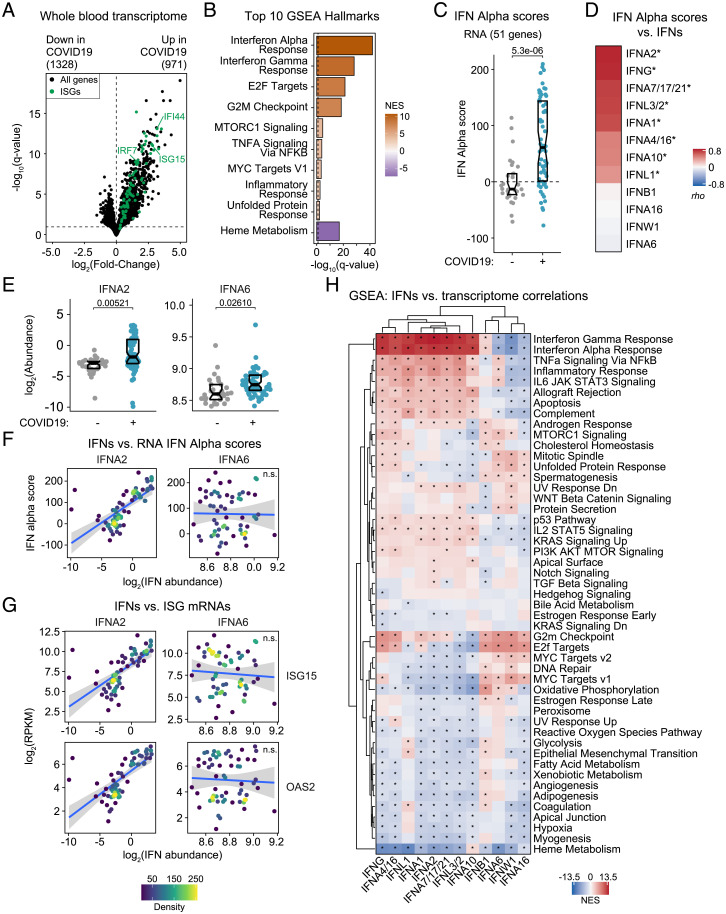
IFN signaling at the whole-blood transcriptome level correlates with a subset of IFNs. (*A*) Volcano plot for differential mRNA expression analysis by COVID-19 status, adjusted for age and sex. Horizontal dashed line indicates a false-discovery rate (FDR) 10% for negative binomial Wald test; numbers above plot indicate significant genes. ISGs are highlighted in green. (*B*) Bar plot of top 10 Hallmark gene sets as ranked by absolute normalized enrichment score (NES) from GSEA. Bar color represents NES; bar length represents -log_10_(*q*-value). (*C*) RNA-based IFN-α scores by COVID-19 status. Data are presented as a modified sina plot with box indicating median and interquartile range; number above bracket is the *q*-value for Mann–Whitney *U* test. (*D*) Ranked heatmap representing correlations between RNA-based IFN-α scores and plasma levels of IFNs. Values are Spearman correlation coefficients (*rho*); asterisks indicate significant correlations (10% FDR). (*E*) Sina plots comparing abundance for the indicated IFNs by COVID-19 status. Data are presented as modified sina plots with boxes indicating median and interquartile range. Numbers above brackets are *q*-values for Mann–Whitney *U* tests. (*F*) Scatter plots showing the relationship between RNA-based IFN-α score and plasma abundance of IFNs in COVID-19 patients. Points are colored by density; blue lines represent linear model fit with 95% confidence intervals in gray. (*G*) Scatter plots showing the relationship between ISG mRNA levels and plasma abundance of IFNs in COVID-19 patients. (*H*) Heatmap representing enrichment of Hallmark gene sets among Spearman correlations between mRNA levels and plasma levels of IFNs. Values displayed are NES from GSEA; asterisks indicate significant enrichment (10% FDR); columns and rows are grouped by hierarchical clustering. See also *SI Appendix*, Figs. S1 and S2. n.s., not significant.

To monitor IFN signaling, we first analyzed the transcriptome dataset. DESeq2 analysis, adjusting for age and sex as covariates, identified 2,299 genes differentially expressed in the blood of COVID-19 patients (Fig. 1*A* and Dataset S2). Gene set enrichment analysis (GSEA) identified the Hallmark Interferon Alpha and Gamma Response gene sets as the most significant positively enriched signatures in COVID-19 patients (Fig. 1*B* and Dataset S3). To assess interindividual variation in expression of these IFN gene signatures, we calculated *z*-score–based IFN-α and -γ scores for each sample, showing that COVID-19 patients display significantly increased yet variable IFN scores relative to controls (Fig. 1*C* and *SI Appendix*, Fig. S1*A*). In order to assess the degree to which this variability in IFN signaling is associated with the levels of circulating IFNs, we mined the SOMAscan proteomics and multiplexed immunoassay datasets (Meso Scale Discovery, MSD), which collectively measured a total of 17 different IFNs. To validate the reagents in these two platforms, we spiked single (SOMAscan) or multiple concentrations (MSD) of commercially available recombinant IFNs into a pooled plasma reference sample (*SI Appendix*, *SI Extended Methods*). We discarded five SOMAscan measurements (IFNA5, IFNA8, IFNA14, IFNA21, IFNL2) due to lack of sensitivity, and relabeled three measurements based on apparent cross-reactivity (IFNA4/16, IFN7/17/21, IFNL3/2) (*SI Appendix*, Fig. S1*B*). When the same IFN was measured by both platforms, we preferred the MSD measurement, which is quantified against a standard curve (*SI Appendix*, Fig. S1*C*). This exercise allowed us to focus on measurements for 12 IFNs in our subsequent analyses: IFNA1, IFNA2, IFNA4/16, IFNA6, IFNA7/17/21, IFNA10, IFNA16, IFNB1, IFNG, IFNL1, INFL3/2, and IFNW1 (*SI Appendix*, Fig. S1 *B*–*D*).

We next determined Spearman correlations between the RNA-based IFN-α scores and levels of these 12 IFNs (Fig. 1*D*). Interestingly, the correlations were highly variable, with four IFNs lacking significant associations with the IFN-α score (IFNB1, IFNA16, IFNW1, and IFNA6). This result is clearly illustrated by the type I subtypes IFNA2 and IFNA6, which are the most and least correlated with IFN-α scores, respectively. Although both subtypes are significantly up-regulated in the plasma of COVID-19 patients (Fig. 1*E*), only IFNA2 levels correlate with the IFN-α scores (Fig. 1*F*) and with mRNA expression of well recognized IFN-inducible genes (ISGs), such as *ISG15* and *OAS2* (Fig. 1*G*). Repeating this analysis for IFN-γ scores produced a similar rank of correlations (*SI Appendix*, Fig. S1*A*).

To explore this phenomenon more deeply, we completed a comprehensive analysis of gene expression signatures in the whole-blood transcriptome associated with varying plasma levels of the 12 IFNs, using only data from COVID-19 patients. Toward this end, we defined Spearman correlations between IFNs and 15,000+ mRNAs detected, which identified thousands of significant correlations, with great variability across IFNs (*SI Appendix*, Fig. S2*A* and Dataset S4). We then analyzed the ranked correlations for each IFN using GSEA to identify known gene sets with significant enrichment among positive or negative correlations (Fig. 1*H* and Dataset S5). This analysis showed that the top gene signatures positively associated with eight of the IFNs are indeed the IFN-α and -γ responses, followed by related inflammatory and immune pathways. In contrast, for the other four IFNs (IFNB1, IFNA6, IFNW1, and IFNA16), the top signatures enriched in the positive correlations are related to cell proliferation, such as G2M checkpoint, E2F targets, and MYC targets (Fig. 1*H*). In fact, some of these IFNs show negative correlations with the IFN-α and -γ responses (Fig. 1*H*). Again, this differential behavior is illustrated by IFNA2 and IFNA6. Whereas mRNAs positively associated with IFNA2 show clear enrichment of the IFN Alpha Response gene set, these same mRNAs are negatively correlated with IFNA6 levels (e.g., *ISG15*) (*SI Appendix*, Fig. S2*B*).

Altogether, these results suggest functional specialization among circulating IFNs, whereby only specific IFNs associate with the IFN transcriptional response of circulating immune cells.

### Individual IFNs Show Differential Proteomic Signatures Associated to COVID-19 Pathology.

**Fig. 2.**
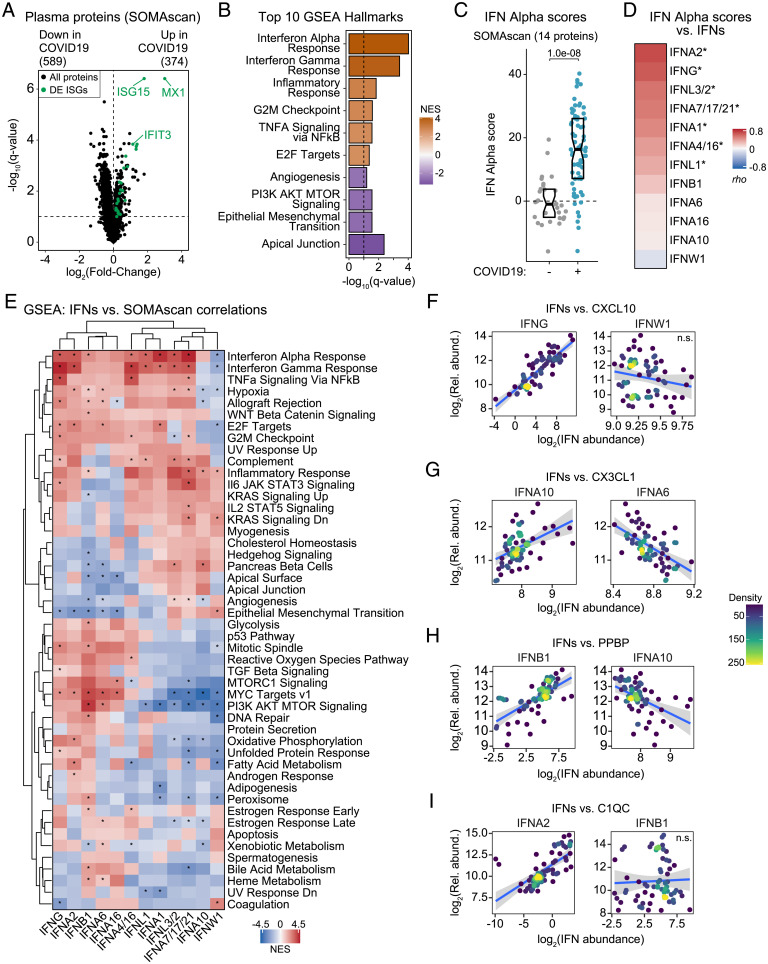
IFN signaling at the proteome level correlates with features of COVID-19 pathophysiology. (*A*) Volcano plot for linear regression analysis of SOMAscan proteomics data by COVID-19 status, adjusted for age and sex. Horizontal dashed line indicates an FDR threshold of 10% (*q* < 0.1); numbers above plot indicate significant proteins. Proteins encoded by ISGs are highlighted in green. (*B*) Bar plot of top 10 Hallmark gene sets as ranked by absolute NES from GSEA. Bar color represents NES; bar length represents -log_10_(*q*-value). (*C*) Protein-based IFN-α scores by COVID-19 status. Data are presented as a modified sina plot with box indicating median and interquartile range. (*D*) Ranked heatmap representing correlations between protein-based IFN-α scores and plasma levels of each IFN. Values displayed are Spearman correlation coefficients (*rho*); asterisks indicate significant correlations (10% FDR). (*E*) Heatmap representing enrichment of Hallmark gene sets among Spearman correlations between plasma levels of proteins measured by SOMAscan versus IFNs. Values displayed are NES from GSEA; asterisks indicate significant enrichment (10% FDR); columns and rows are grouped by hierarchical clustering. (*F*–*I*) Scatter plots comparing relationships between plasma proteins and IFNs in COVID-19 patients. Points are colored by density; blue lines represent linear model fit with 95% confidence intervals in gray. See also *SI Appendix*, Figs. S3 and S4. n.s., not significant.

Next, we investigated the proteomic signatures associated with each IFN. Using linear regression, adjusting for age and sex, we identified 963 epitopes measured by SOMAscan differentially abundant in the plasma of COVID-19 patients (Fig. 2*A* and Dataset S6). GSEA identified Hallmark IFN alpha and gamma responses as the top proteomic signatures induced in COVID-19 (Fig. 2*B* and Dataset S7). As for the transcriptome, we calculated protein-based IFN-α and -γ scores for each sample, which showed significantly higher yet variable IFN scores among COVID-19 patients (Fig. 2*C* and *SI Appendix*, Fig. S3*A*). Notably, protein-based IFN scores may inform about the organismal IFN response, not just that of circulating immune cells driving the whole-blood transcriptome IFN signature, as multiple organs and tissues could contribute to secretion of IFN-related proteins.

We then defined correlations between the 12 IFNs and the protein-based IFN scores, which revealed both similarities and differences relative to the RNA-based IFN scores (Fig. 2*D* and *SI Appendix*, Fig. S3*A*). Whereas IFNA2 and IFNG remained the most correlated with the protein-based IFN-α and -γ scores, other IFNs behaved differently (Fig. 2*D* and *SI Appendix*, Fig. S3*A*). For example, IFNA10, which was significantly correlated with the RNA-based IFN scores, was not so with the protein-based scores. In contrast, IFNA6 and IFNB1 ranked higher in their association with the protein-based scores (Figs. 1*D* and 2*D* and *SI Appendix*, Figs. S1*A* and S3*A*). We then defined Spearman correlations between the 12 IFNs and 4,800+ epitopes measured by SOMAscan and analyzed the correlation results with GSEA (Fig. 2*E*, *SI Appendix*, Fig. S3*B*, and Datasets S8 and S9). Interestingly, some IFNs with weak transcriptome signatures present strong proteomic signatures. For example, IFNA6 and IFNB1, which show very weak correlations with mRNAs (*SI Appendix*, Fig. S1*A*), are among the IFNs with the most numerous significant associations with circulating proteins (*SI Appendix*, Fig. S3*B*). This suggests that whereas IFNA6 and IFNB1 may not contribute to the IFN transcriptional response of circulating immune cells, they may nonetheless contribute to IFN responses in tissues and organs contributing to the protein-based plasma IFN signature. This is illustrated by the behavior of CXCL11, a canonical ISG, which is significantly correlated at the protein level with IFNA2, IFNA6, and IFNB1 (*SI Appendix*, Fig. S4*A*). Additionally, IFNs often display highly dissimilar, even opposite, relationships to certain proteomics signatures, as illustrated by the PI3K/AKT/mTOR signature (Fig. 2*E*; compare correlations to HRAS for IFNA1, IFNA6, and IFNB1 in *SI Appendix*, Fig. S4*B*).

To probe further into this phenomenon, we examined the top five positively and negatively correlated epitopes for each IFN using unsupervised clustering analysis, which revealed many specialized relationships of relevance to COVID-19 pathophysiology (*SI Appendix*, Fig. S4*C*). For example, several chemokines involved in immune control showed differential associations, such as CXCL10 (compare IFNG to IFNW1 in Fig. 2*F*), CX3CL1 (compare IFNA10 to IFNA6 in Fig. 2*G*), CCL7 (compare IFNA2 to IFNA16 in *SI Appendix*, Fig. S4*D*), and CCL5 (compare IFNB1 to IFNA10 in *SI Appendix*, Fig. S4*E*). Notably, the top positive correlations for IFNB1 are dominated by proteins stored in α-granules of platelets, such as PPBP (multiple SOMAscan aptamers), PDGFA, PDGFD, and PF4 (*SI Appendix*, Fig. S4*C* and Dataset S8). These markers of platelet degranulation are also associated, albeit to a lesser degree, with IFNA6, but not so with other IFNs (Fig. 2*H*, compare IFNB1 to IFNA10, and *SI Appendix*, Fig. S4*C*). This suggests that IFNB1 production is associated with platelet activation, which could be interpreted as a sign of endothelial damage at sites producing IFNB1. A subset of IFNs showed strong associations with complement factors, such as C1QC (Fig. 2*I*, compare IFNA2 to IFNB1, and *SI Appendix*, Fig. S4*C*). The top correlated epitope for IFNA10 is TRIL, a component of the Toll-like receptor-4 complex, but this association was weaker for many other IFNs (*SI Appendix*, Fig. S4*C*, compare IFNA10 to IFNA6 in *SI Appendix*, Fig. S4*F*). KIR3DL2 and KIR3DS1, two killer cell immunoglobulin (Ig)-like receptors expressed by natural killer (NK) cells and subtypes of T cells, were strongly correlated with a subset of IFNs, most prominently IFNA6 (*SI Appendix*, Fig. S4*C*, compare IFNA6 to IFNA10 in *SI Appendix*, Fig. S4*G*). OLFM4 (Olfactomedin 4), a protein selectively expressed in inflamed colonic epithelium, was strongly associated with IFNA4/16, but not other IFNs (*SI Appendix*, Fig. S4*C*, compare IFNA4/16 versus IFNA10 in *SI Appendix*, Fig. S4*H*).

Altogether, these results reveal that circulating levels of different IFNs associate with proteomic signatures indicative of diverse pathophysiological processes, such as tissue-specific inflammation, complement activation, and endothelial damage.

### Differential IFN Action at the Cell-Based vs. Organismal Levels.

**Fig. 3.**
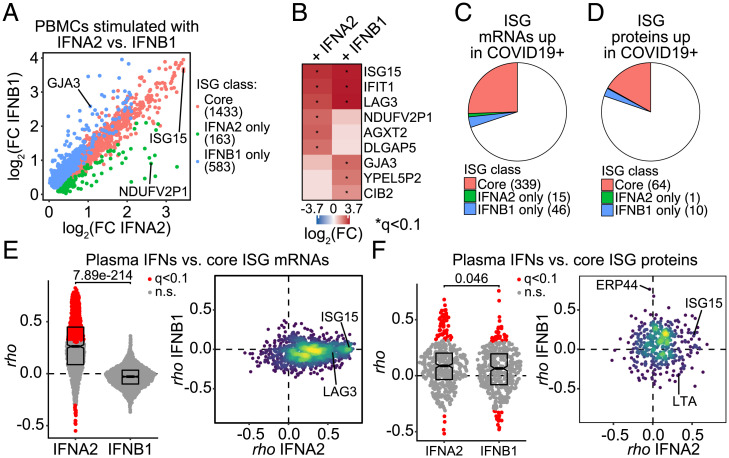
The cellular action of IFNA2 and IFNB1 does not explain their differential biosignatures in COVID-19. (*A*) Scatter plot comparing fold-changes for IFNA2- and IFNB1-stimulated genes in PBMCs treated ex vivo. (*B*) Heatmap representing differential expression of selected genes from each class in *A*. Values displayed are fold-changes for stimulation with IFNA2/baseline and IFNB1/baseline; asterisks indicate significant differences over baseline (10% FDR); rows are grouped by hierarchical clustering. (*C* and *D*) Pie charts displaying the relative fraction of mRNAs (*C*) or proteins (*D*) up-regulated in COVID-19 patients in each class from *A*. Absolute numbers are indicated in legend. (*E*) Spearman correlation score (*rho*) distributions for core mRNAs against plasma levels of IFNA2 and IFNB1. Data are presented as a modified sina plot with boxes indicating median and interquartile range with number above bracket indicating the *q*-value for Mann–Whitney *U* test (*Left*) and scatter plots with points colored by density (*Right*). (*F*) Spearman correlation score (*rho*) distributions for core proteins detected by SOMAscan against plasma levels of IFNA2 and IFNB1. Data are presented as a modified sina plot with boxes indicating median and interquartile range with number above bracket indicating the *q*-value for Mann–Whitney *U* test (*Left*) and scatter plot with points colored by density (*Right*). n.s., not significant.

Up to this point, our analyses indicate specialized IFN action in the context of SARS-CoV-2 infection, which could be explained by several nonmutually exclusive potential mechanisms, including tissue-specific expression of IFNs (22), variable timing of IFN production during the course of viral infection (23), self-amplification of certain IFNs via positive feedback (24, 25), differential turnover of IFNs in circulation (26), or blockade of specific IFN subtype production by SARS-CoV-2 proteins (27). Remarkably, this specialization was different when analyzing the transcriptome versus proteome. To investigate this phenomenon further, we stimulated peripheral blood mononuclear cells (PBMCs) from uninfected donors with IFNA2 and IFNB1, two type I subtypes that show distinct relationships with the transcriptome of COVID-19 patients, and analyzed their impact on the PBMC transcriptome at 18 h posttreatment (*SI Appendix*, *SI Extended Methods*) (28). We identified 2,179 RNAs significantly induced by at least one subtype, with 65.6% being induced by both IFNs (core ISGs), 7.4% of them being significantly induced by IFNA2 only (173 genes), and 26.7% by IFNB1 only (583 genes) (Fig. 3*A*). Examples of core ISGs are ISG15, IFIT1, and LAG3; examples of IFNA2-specific ISGs are AGX2, DLGAP5, and NDUFVP2; and examples of IFNB1-specific ISGs are GJA3, YPEL5P2, and CIB2 (Fig. 3*B*). Notably, ∼25% of all mRNAs induced in the whole-blood transcriptome of COVID-19 patients correspond to core ISGs identified in PBMCs (Fig. 3*C*) and, at the protein level, ∼17% of proteins elevated in COVID-19 patients are encoded by core ISGs (Fig. 3*D*). Thus, type I IFN is a major contributor to the transcriptome and proteome changes observed in COVID-19. However, analysis of correlations between circulating levels of the two subtypes indicates that only IFNA2 levels are significantly correlated with mRNA expression of core ISGs (Fig. 3*E*). Of the core ISG mRNAs detected in the whole-blood transcriptome, ∼45% have significant positive correlations with IFNA2, whereas none have significant correlations with IFNB1. In contrast, at the protein level, both subtypes display numerous significant correlations with proteins encoded by core ISGs (Fig. 3*G*).

Altogether, these results indicate that the differential relationship between ISG expression in circulating blood cells and levels of IFNA2 and IFNB1 is not necessarily a function of their ability to induce transcription of core ISGs, but rather the consequence of other mechanisms acting at the organismal level.

### Seroconversion Modulates the Peripheral IFN Milieu.

**Fig. 4.**
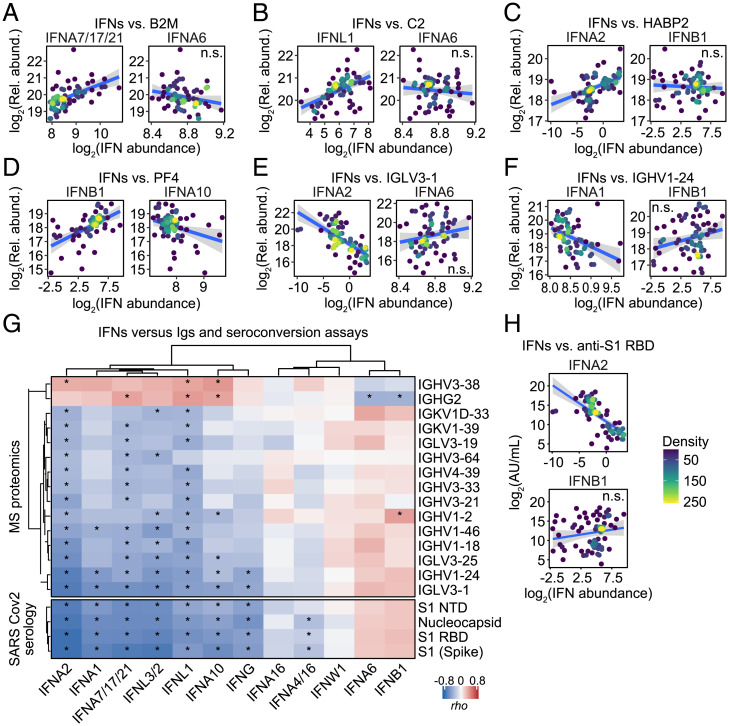
Differential association of IFNs with seroconversion. (*A*–*F*) Scatter plots comparing relationships between plasma proteins measured by MS proteomics and IFNs in COVID-19 patients. Points are colored by density; blue lines represent linear model fit with 95% confidence intervals in gray. (*G*) Heatmap representing correlations between IFNs and plasma levels of Igs measured by MS proteomics (*Upper*), or antibody reactivity against SARS-CoV-2 measured by immunoassays (*Lower*). Values displayed are Spearman correlation scores (*rho*); asterisks indicate significant correlations (10% FDR); columns and rows are grouped by hierarchical clustering. (*H*) Scatter plots comparing relationships between plasma antibody reactivity against SARS-CoV-2 S1 RBD region and IFNA2/B1 in COVID-19 patients. Points are colored by density; blue lines represent linear model fit with 95% confidence intervals in gray. See also *SI Appendix*, Figs. S5 and S6. n.s., not significant.

Next, we analyzed correlations between the 12 IFNs and plasma proteins measured by our MS platform, which is complementary to the SOMAscan dataset as it detects many abundant proteins for which SOMAmer reagents are not available, including various Igs. Using linear regression, adjusting for age and sex, we identified 70 proteins differentially abundant in plasma of COVID-19 patients (*SI Appendix*, Fig. S5*A* and Dataset S11). Of the 28 significantly elevated proteins, 17 of them are Igs (labeled green in *SI Appendix*, Fig. S5*A*), potentially indicative of seroconversion in some patients. We then calculated Spearman correlations between IFNs and all proteins detected by MS (*SI Appendix*, Fig. S5*B* and Dataset S12) and visualized the top five positively and negatively correlated proteins for each IFN via unsupervised hierarchical clustering (*SI Appendix*, Fig. S6*A*). This analysis confirmed some observations made with the SOMAscan dataset, but also revealed several new associations. First, a subset of IFNs associates strongly with IFN-inducible proteins, such as B2M (compare IFNA7/17/21 to IFNA6 in Fig. 4*A*), and LGALS3BP (compare IFNA4/16 to IFNA6 in *SI Appendix*, Fig. S6*B*). Second, many of the same IFNs associate with elevated levels of complement subunits, such as C2 (compare IFNL1 to IFNA6 in Fig. 4*B*) and C9 (compare IFNA2 to IFNA6 in *SI Appendix*, Fig. S6*C*). Third, several key regulators of coagulation and fibrinolysis were significantly associated with specific IFNs. Salient examples include HABP2 (compare IFNA2 to IFNB1 in Fig. 4*C*), FGA (compare IFNL1 to IFNA16 in *SI Appendix*, Fig. S6*D*), F13B (compare IFNA10 to IFNA6 in *SI Appendix*, Fig. S6*E*), and PROZ (compare IFNW1 to IFNB1 in *SI Appendix*, Fig. S6*F*). Fourth, very distinctly, IFNB1—and to a lesser degree IFNA6—associate positively with markers of platelet degranulation, such as PF4, THBS1, PPBP, MMRN1, and SPARC (Fig. 4*D*, compare IFNB1 to IFNA10, and *SI Appendix*, Fig. S6*A*). Finally, IFNs have distinct relationships to a subset of Ig heavy- and light-chain variable domain peptides that were associated positively or negatively with the levels of specific IFNs (compare IFNA2 to IFNA6 in Fig. 4*E* and IFNA1 to IFNB1 in Fig. 4*F*). This result could be explained by varying levels of IFNs upon seroconversion (20).

To investigate in detail the interplay between specific IFNs, Ig levels, and seroconversion, we examined correlations between the IFNs and all Ig variable domains detected by MS proteomics, as well as seroconversion assays used to detect IgGs against SARS-CoV-2 peptides (S1 full-length, spike; S1 N terminus; and S1 receptor binding domain [RBD]; nucleocapsid) (Fig. 4*G*). This analysis revealed that a subset of IFNs is strongly anticorrelated with seroconversion (e.g., compare IFNA2 to IFNB1 in Fig. 4*H*) and specific Ig variable domains that have been previously found enriched in the bloodstream of COVID-19 patients, such as IGHV1-24 and IGLV3-1 (29). This observation could be interpreted as early production of some IFNs with subsequent declines upon seroconversion (e.g., IFNA2, IFNG), followed by later production of other IFNs (e.g., IFNA6, IFNB1), potentially from sites where SARS-CoV-2 evades humoral neutralization. Overall, these results further support the notion of differential action of IFNs in COVID-19 pathophysiology, suggesting a temporal sequence of IFN production before and after seroconversion.

### Distinct Immune Cell Signatures Associate with Fluctuations in IFN Levels.

**Fig. 5.**
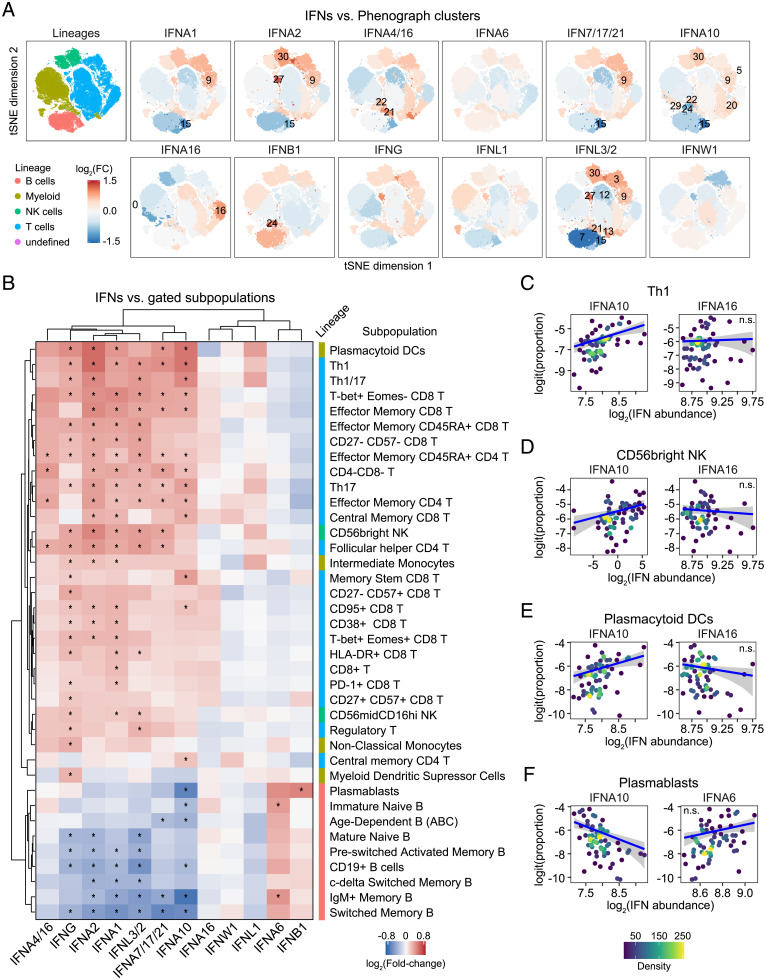
Differential association of IFNs with immune cell signatures. (*A*) t-SNE plots of 69,000 cells analyzed by mass cytometry from 69 COVID-19 patients (1,000 cells each). Leftmost panel is colored by major cell lineages; all other panels have cells within each PhenoGraph cluster (as shown in *SI Appendix*, Fig. S7*A*) colored by the fold-change in cluster proportion among live cells per SD of abundance for the indicated IFN, as determined by β-regression analysis, adjusting for age and sex; numbers indicate clusters with significant associations with IFN abundance (10% FDR). (*B*) Heatmap representing relationships between IFNs and gated subpopulation proportions among live cells, as determined by β-regression analysis. Values displayed are fold-change in cluster proportion among live cells per SD of IFN abundance; asterisks indicate significant associations (10% FDR); columns and rows are grouped by hierarchical clustering. (*C*–*F*) Scatter plots comparing relationships between gated subpopulation proportions among live cells and IFNs in COVID-19 patients. Points are colored by density; blue lines represent β-regression model fit with 95% confidence intervals in gray. See also *SI Appendix*, Figs. S7 and S8. n.s., not significant.

Next, we investigated the relationship between plasma levels of IFNs and circulating immune cells analyzed by mass cytometry. First, we employed the unsupervised clustering algorithm PhenoGraph (30) to identify distinct subpopulations of immune cells, combined with t-stochastic neighbor embedding (t-SNE) dimensionality reduction to aid in visualization (31), resulting in identification of ∼30 clusters (Fig. 5*A* and *SI Appendix*, Fig. S7 *A* and *B***)**. We then identified clusters whose relative frequency among all live cells was significantly associated with varying IFN levels, using β-regression, with adjustment for age and sex (Fig. 5*A*, *SI Appendix*, Fig. S7 *C* and *D*, and Dataset S13). This analysis revealed that multiple IFNs are significantly associated with increased abundance of clusters enriched for T cells and NK cells, while also displaying negative associations with clusters enriched for B cells (Fig. 5*A* and *SI Appendix*, Fig. S7 *C* and *D*). For example, IFNA1 is positively associated with clusters 9 (CD8^+^ T cells) and 30 (CD56^+^ NK cells) and negatively associated with cluster 15 (switched memory B cells) (*SI Appendix*, Fig. S7*E*).

To further these observations in relationship to known immune cell subpopulations, we analyzed associations between the IFNs and 50+ immune cell types defined by traditional gating based on marker expression (Fig. 5*B*, *SI Appendix*, Fig. S8*A*, and Datasets S14 and S15). This exercise confirmed specialized relationships between some IFNs and specific lymphoid cell subsets. For example, among CD4^+^ T cells, the T-helper 1 (Th1) subset displays significant positive associations only with IFNA1, IFNA2, IFNA7/17/21, IFNA10, IFNG, and IFNL3/2 (Fig. 5*B*, compare IFNA10 to IFNA16 in Fig. 5*C*). This pattern was also apparent for many, but certainly not all, T cell subsets (Fig. 5*B*). Similarly, NK CD56^bright^ cells also showed differential positive relationships with IFNs, with an overall pattern similar to that of key T cell subsets (compare IFNA2 to IFNA16 in Fig. 5*D*). Notably, this analysis also revealed significant positive associations between specific IFNs and plasmacytoid dendritic cells (DCs), which are strong producers of IFNs during viral infections (compare IFNA10 to IFNA16 in Fig. 5*E*). Many IFNs positively associated with CD4^+^ T cell subsets were negatively associated with B cell subsets, while IFNA6 displays the opposite relationship (Fig. 5*B*, compare IFNA10 versus IFNA6 in Fig. 5*F*). These differential associations could be interpreted as a transition from T cell-driven responses prior to seroconversion, followed by B cell activation and differentiation toward antibody-producing plasmablasts during seroconversion, along with decreased production of a specific subset of IFNs.

Altogether, these results suggest a temporal sequence of IFN production in coordination with changes in the peripheral immune cell compartment. An overview of salient IFNs associations along the paths of T cell and B cell activation and differentiation is shown in *SI Appendix*, Fig. S8*B*.

### Metabolic Signatures of IFN Signaling in COVID-19.

**Fig. 6.**
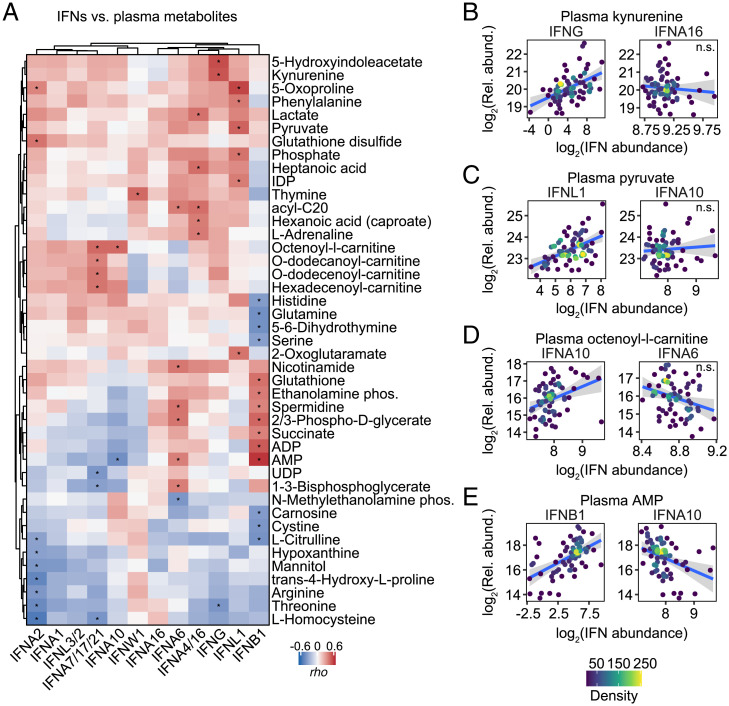
Differential metabolic signatures associated with IFNs. (*A*) Heatmap representing correlations between IFNs and plasma metabolites. Values displayed are Spearman correlation scores (*rho*); asterisks indicate significant correlations (10% FDR); columns and rows are grouped by hierarchical clustering. (*B*–*E*) Scatter plots comparing relationships between select metabolites and IFNs in COVID-19 patients. Points are colored by density; blue lines represent linear model fit with 95% confidence intervals in gray. See also *SI Appendix*, Figs. S9 and S10. n.s., not significant.

Next, we investigated metabolic signatures associated with varying levels of IFNs, calculating Spearman correlations for detected metabolites in plasma and RBC samples against each of the IFNs (*SI Appendix*, Fig. S9 and Datasets S16 and S17). In plasma, significant positive correlations were observed between the tryptophan/indole pathway metabolites kynurenine and 5-hydroxyindoleacetate and IFNG, but not other IFNs (Fig. 6*A*, compare IFNG to IFNA16 in Fig. 6*B*). In RBCs, kynurenine pathway metabolites showed a strong positive association with IFNG, as well as IFNA7/17/21 (*SI Appendix*, Fig. S10 *A* and *B*). Activation of the kynurenine pathway has documented in COVID-19 (32), and kynurenine production can be stimulated by induction of IDO1, an ISG downstream of all three major types of IFN signaling (33). Therefore, it is interesting that this pathway is preferentially associated with IFNG in COVID-19.

Plasma levels of IFNA2 showed significant positive correlations with the markers of oxidative stress glutathione disulfide and 5-oxoproline, a byproduct of the γ-glutamyl cycle (Fig. 6*A*, compare IFNA2 to IFNW1 in *SI Appendix*, Fig. S10*C*), and negatively associated with markers of endothelial dysfunction and nitric oxide signaling (arginine, citrulline) (Fig. 6*A*; compare IFNA2 to IFNW1 in *SI Appendix*, Fig. S10*D*). In RBCs, IFNA2 once again had strong positive correlations with several markers of oxidative stress (5-oxoproline) or pentose phosphate pathway activation (sedoheptulose phosphate) (*SI Appendix*, Fig. S10 *A* and *E*), which is required in RBCs to generate reducing equivalent (NADPH) for recycling oxidized glutathione and other NADPH-dependent antioxidant enzymes. IFNA2 levels also positively correlated with fatty acid mobilization in RBCs (*SI Appendix*, Fig. S10*A*), likely a result of the activity of peroxiredoxin 6 or phospholipase A2 activity on complex lipids to fuel fatty acid release in the bloodstream to sustain viral capsid formation (34). Of note, among the positive correlates to IFNA2 levels in the fatty acid compartment, we observed only saturated (octanoic, dodecanoic, hexadecanoic, octadecanoic) or monounsaturated fatty acids (tetradecenoic, hexadecenoic, octadecenoic) (compare IFNA2 to IFNB1 in *SI Appendix*, Fig. S10*F*), suggestive of limited fatty acid desaturase activation despite the stress induced by the viral infection (35). Several ATP precursors/breakdown products (AMP and adenine) positively correlated with IFNA2 in RBCs, as did pyruvate, phosphate and diphosphate, all suggestive of altered glycolysis and overall energetics associated with IFNA2 signaling (*SI Appendix*, Fig. S10*A*). IFNA2 also negatively correlated with several amino acids in RBCs, including the antioxidants taurine, arginine, threonine and methionine, critical for RBC redox damage repair in the face of the incapacity to synthesize new proteins (*SI Appendix*, Fig. S10*A*) (36).

Plasma IFNL1 significantly correlated with several glycolytic metabolites (e.g., pyruvate) (compare IFNL1 to IFNA1 in Fig. 6*C*), as well as short-chain fatty acids hexanoate and heptanoate, potentially indicative of dysregulation of mitochondrial metabolism in patients with high IFNL1. In RBCs, IFNL1 levels showed positive correlations with the levels of inosine diphosphate (IDP) (compare IFNL1 to IFNA6 in *SI Appendix*, Fig. S10*G*) and negative correlations with carnitine and acetylcarnitine, potentially suggestive of RBC deformability issues (37) as a function of IFNL1 signaling.

Plasma IFNA7 and IFNA10 (and to a lesser extent IFNA1 and IFNA2) were positively associated with a cluster of acylcarnitines (including octenoyl, dodecanoyl, dodecenoyl, hexadecenoyl-carnitine) (compare IFNA10 to IFNA6 in Fig. 6*D*), suggesting an association between these IFNs and altered fatty acid oxidation. These data are relevant in light of the role of acylcarnitines in coagulation and the dysregulation of coagulation cascades in COVID-19 (38).

Plasma levels of IFNB1 showed a strong negative correlation with metabolites tied to the nitric oxide pathway (citrulline), as well as other amine group donors (glutamine, serine) or oxidant stress-related metabolites (carnosine, cystine). On the other hand, IFNB1 positively correlated with the plasma levels of glutathione and spermidine (antioxidant metabolites), succinate (marker of mitochondrial dysfunction), and purinergic agonists involved in vasodilatory/hypoxic responses (ADP and AMP), perhaps produced by hemolytic events (compare IFNB1 to IFNA10 in Fig. 6*E* and *SI Appendix*, Fig. S10 *H* and *I*).

Altogether, these results not only confirm metabolic signatures previously associated with IFN signaling (e.g., activation of the kynurenine pathway), but also reveal unexpected associations between specific IFNs and diverse metabolic processes dysregulated in COVID-19.

### Interplay between IFNs and Markers of COVID-19 Risk and Severity.

**Fig. 7.**
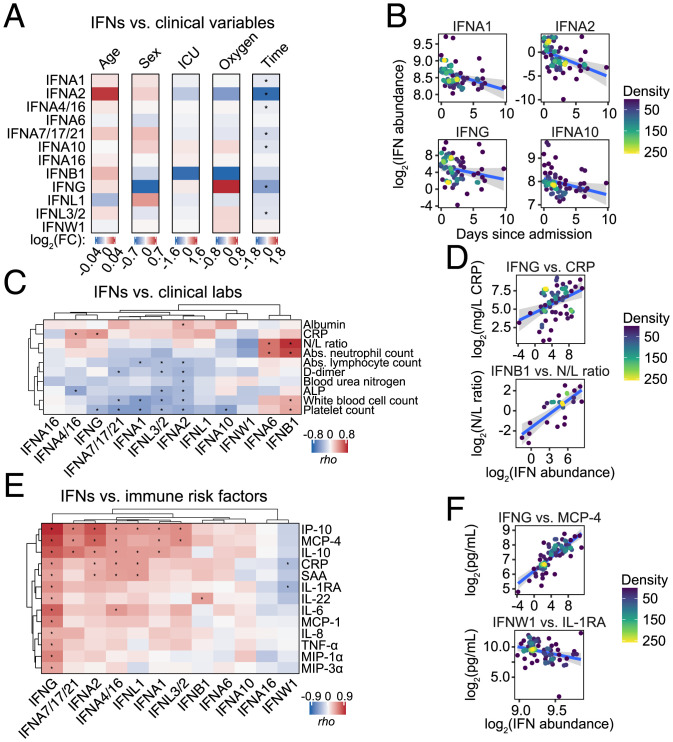
Differential association of IFNs with clinical variables and markers of prognosis in COVID-19. (*A*) Heatmaps summarizing linear regression analysis of plasma IFNs abundance against age (continuous), sex (males/females), ICU status (ever ICU/never ICU), O_2_ group (high/low), or days since admission (continuous, limited to ≤14 d) for COVID-19^+^ samples, adjusted for age and/or sex as appropriate; asterisks indicate significant associations (10% FDR). (*B*) Scatter plots comparing relationships between days since admission and IFNs in COVID-19 patients. Points are colored by density; blue lines represent linear model fit with 95% confidence intervals in gray. (*C*) Heatmap representing correlations between clinical laboratory measurements and plasma levels of each IFN. Values displayed are Spearman correlation coefficients (*rho*); asterisks indicate significant correlations (10% FDR). (*D*) Scatter plots comparing relationships between clinical laboratory measurements and the indicated IFNs in COVID-19 patients. (*E*) Heatmap representing correlations between selected immune factors associated with poor prognosis in COVID-19 measured in plasma by MSD assays and plasma levels of IFNs. Values displayed are Spearman correlation coefficients (*rho*); asterisks indicate significant correlations (10% FDR). (*F*) Scatter plots comparing relationships between immune factors and IFNs in COVID-19 patients.

Next, we aimed to define the relationship between IFNs and available clinical variables, using linear regression with adjustment for age and sex. Among COVID-19 patients, none of the IFNs was significantly different by age or sex (Fig. 7*A* and Dataset S18). Although all samples in this study were collected when patients were presenting mild-to-moderate symptomology, some patients were subsequently admitted to an intensive care unit (ICU) or required higher O_2_ supplementation. None of the IFNs was significantly different by ICU status (i.e., never ICU vs. ever ICU) or O_2_ requirement (high vs. low) (*Methods*). However, seven IFNs showed significant decreases with increasing time between hospital admission and research blood draw (Fig. 7 *A* and *B*), all of which were negatively correlated with seroconversion values (Fig. 4*B*). This indicates that whereas specific IFNs are produced earlier in the course of symptomology, prior to seroconversion, others are produced later in the course of the disease, a notion supported by longitudinal analyses (3, 8, 39, 40).

Next, we investigated correlations between IFNs and clinical laboratory values closest in time to the research blood draw, which revealed several significant associations (Fig. 7*C* and Dataset S19). For example, IFNG and IFNA4/16 were the most positively correlated with levels of C-reactive protein (CRP), a well-recognized biomarker of poor prognosis at the time of hospitalization (Fig. 7 *C* and *D*) (41). In contrast, IFNB1 and IFNA6 were significantly correlated with the neutrophil/lymphocyte ratio (N/L ratio), a marker of severe COVID-19 pathology (42) (Fig. 7 *C* and *D*). Several IFNs were significantly associated with depletion of white blood cells, lymphocytes, and platelets (Fig. 7*C*), most of which also showed decreased levels with time since hospitalization (Fig. 7 *A* and *B*), supporting the notion that cytopenias occur earlier in the course of COVID-19, prior to seroconversion (20). Notably, IFNB1 (and to a lesser degree IFNA6) showed positive correlations with platelet numbers, suggesting that platelet recovery later in the pathological cascade co-occurs with increased levels of these IFNs (Fig. 7*C*).

Next, we investigated more deeply the interplay between IFNs and immune markers whose elevation in circulation has been associated with poor prognosis in COVID-19, including interlelukin (IL)-22, SAA, IL-10, IP-10, IL-6, IL-8, MIP-3α, IL-1RA, MIP-1α, TNF-α, MCP-1, and MCP-4 (Fig. 7*E* and Dataset S20) (39, 40, 43–45). This analysis highlighted IFNG as the IFN with the highest number of positive associations with these immune markers of poor prognosis (Fig. 7 *E* and *F*). Overall, each IFN has its unique profile of association with these markers, as clearly evidenced by IFNB1, which is strongly positively associated only with IL-22, or IFNW1, which has negative correlations with CRP and IL1-RA (Fig. 7 *E* and *F*).

Altogether, these observations reveal differential relationships between different IFNs and clinical variables in COVID-19, with some IFNs being associated with the hyperinflammatory stage of the disease (e.g., IFNG), whereas others associate with markers of late severe disease, such as increased N/L ratio (e.g., IFNB1).

## Discussion

IFN signaling is a critical component of the innate immune response and a main driver of the antiviral defense. In the context of viral infections, deficiencies in IFN signaling cause profound susceptibility in humans, as demonstrated by various inborn errors of immunity affecting IFN signaling (46). Despite these protective effects, exacerbated IFN signaling can also contribute to diverse pathologies, as exemplified by type I interferonopathies (47). In the context of COVID-19, the role of IFN signaling has been the subject of much study and debate, with both protective and deleterious effects being documented in different experimental systems and clinical settings (4–6, 8, 9, 11–13, 19, 40). Within this framework, we completed a comprehensive analysis of multiomics signatures associated with production of multiple IFNs in hospitalized COVID-19 patients, revealing a high degree of diversity, even among closely related subtypes.

During vertebrate evolution, the IFN gene family has undergone significant expansion through tandem gene duplication and retrotransposition events, likely contributing to increased regulatory diversity and functional specialization (1). Although modest, our current understanding of IFN specialization is increasing. Functional specialization between major type I, II, and III IFNs has been revealed by analyzing genetic mutations affecting specific receptors or downstream kinases and transcription factors in both humans and mice (1, 46). For example, it is accepted that deficiencies in type I/III signaling confer susceptibility to viral infections, whereas deficiencies in IFNG signaling are associated with mycobacterial disease (46). IFN specialization is also evident in the clinical use of recombinant subtypes, with IFNB1 being the most effective for the treatment of multiple sclerosis, whereas IFNA2 preparations are preferred for the treatment of chronic viral infections and some malignancies (48). Notably, type I subtypes display vastly different potencies to impede HIV replication ex vivo (49, 50), and SARS-CoV-2 replication in vitro (51), with different constellations of subtypes being the most effective to control each virus. Despite these advances, little is known about the mechanisms behind these differential effects (52). In this context, our work provides a valuable resource for future mechanistic research.

Although our multiomics analysis is descriptive in nature and based largely on statistically significant associations that should not be interpreted as cause–effect relationships, its value is confirmed by the many associations observed for which mechanisms have already been established. For example, our unbiased analysis of the transcriptome confirmed that 8 of the 12 IFNs tested are indeed significantly associated with a transcriptional program highly enriched for ISGs. Likewise, the association between IFNG and metabolites in the kynurenine pathway can be explained by induction of IDO1, a known ISG, during the inflammatory response elicited by SARS-CoV-2 (32, 33). Therefore, using these confirmatory observations as reference points, we propose that the datasets described here will help the field elucidate many novel cause–effect relationships explaining IFN specialization.

The specialized biosignatures of IFN action could be due to several nonmutually exclusive mechanisms, such as action through different receptors (1), differences in affinity or allosteric regulation for the same receptors (1, 52), differences in the location and timing of IFN production (23), differential turnover in circulation of various IFNs (26), differential self-amplification of IFNs (24, 25), or uneven antagonism of IFN production by SARS-CoV-2 proteins (27). One limitation of our study is that all measurements were performed from peripheral blood, which can only inform about a subset of the pathophysiological processes modulated by various IFNs. However, other studies have documented differences in IFN action and regulation at different sites during SARS-CoV-2 infection, even along the upper and lower respiratory tract (8, 53), or when comparing mucosal versus systemic immune responses (54). It is also possible that the specialized biosignatures observed are driven in part by SARS-CoV-2 itself. Like other members of the coronavirus family, SARS-CoV-2 has evolved diverse strategies to evade the antiviral effects of IFN signaling, and it is likely that these escape mechanisms do not affect all IFNs equally (27).

Despite these limitations, key observations produced by our study include the differential relationship between IFNs and the antiviral transcriptional program in circulating immune cells, the specialized relationship between seroconversion, immune cell-type abundance and IFN levels, distinct metabolic signatures associated with each IFN, and their differential relationship with clinical metadata and biomarkers of poor prognosis and severity. Throughout the study, the contrast between IFNA2 and IFNA6 exemplifies these points. Both IFNA2 and IFNA6 are significantly up-regulated in the COVID-19^+^ cohort. However, whereas IFNA2 is strongly associated with the IFN transcriptional program in immune cells, IFNA6 is not. IFNA2 proteomic signatures are enriched for cytokines and chemokines previously linked to IFN signaling, whereas IFNA6 proteomic signatures, similarly to those of IFNB1, are enriched for markers of platelet degranulation. IFNA2 levels decrease with seroconversion and time since hospitalization; IFNA6 levels do not. Accordingly, IFNA2 abundance associates with increased frequency of various T cell subsets involved in the early antiviral response, while IFNA6 levels correlate with B cell maturation. While IFNA2 has the highest number of significant associations in the RBC metabolome, IFNA6 has none. Lastly, only IFNA2 levels correlate with many immune markers of poor prognosis. Therefore, a detailed comparative study of these two IFNA subtypes is warranted, including studies in human cell preparations and animal models.

In sum, our analyses and datasets provide a rich resource to advance understanding of the IFN family in humans. To accelerate the use of these datasets, they are made readily available through the COVIDome Explorer Researcher Portal (21), where users can recreate the cross-omics correlations described here, investigate any other cross-omics correlations of choice, and download all data for further analysis.

## Methods

### Study Design, Participant Recruitment, and Clinical Data Capture.

Research participants were recruited and consented for participation in the COVID Biobank of the University of Colorado Anschutz Medical Campus (Colorado Multiple Institutional Review Board [COMIRB] Protocol #20-0685). Data were generated from deidentified biospecimens and linked to demographics and clinical metadata procured through the Health Data Compass data warehouse at the University of Colorado under COMIRB Protocol #20-1700. Participants were hospitalized either at Children’s Hospital Colorado or the University of Colorado Hospital. COVID-19 status was defined by a positive PCR result or antibody test within 14 d of the research blood draw. The control cohort consisted of COVID-19^−^ research participants receiving medical care for a range of conditions, none of them in critical condition at the time of the research blood draw. ICU status was defined by whether the patient was subsequently admitted to an ICU, but all research blood draws were obtained prior to any such events. Respiratory support (O_2_ group) was defined using the highest level of oxygen support required during the entire hospital stay, with “low” consisting of room air or oxygen mask only, and “high” consisting of noninvasive ventilation, high-flow nasal canula, heated high-flow nasal canula, or invasive ventilation. A summary of cohort characteristics can be found in Dataset S1.

### Dataset Generation and Primary Analysis.

Methods for blood processing, whole-blood RNA library preparation and sequencing, plasma proteomics by MS and SOMAscan assays, cytokine profiling and seroconversion by multiplex immunoassay, mass cytometry analysis, and MS-based metabolomics of plasma and RBC have been described previously (20, 21) and are provided in full in *SI Appendix*, *SI Extended Methods*.

## Supplementary Material

Supplementary File

Supplementary File

Supplementary File

Supplementary File

Supplementary File

Supplementary File

Supplementary File

Supplementary File

Supplementary File

Supplementary File

Supplementary File

Supplementary File

Supplementary File

Supplementary File

Supplementary File

Supplementary File

Supplementary File

Supplementary File

Supplementary File

Supplementary File

## Data Availability

Anonymized RNA-seq data reported in this paper have been deposited in the Gene Expression Omnibus (GEO) database, https://www.ncbi.nlm.nih.gov/geo (accession nos. GSE167000 and GSE191317). Meso Scale Discovery platform cytokine profiling has been deposited in Mendeley, https://data.mendeley.com/datasets/2mc6rrc5j3/2. SOMAscan proteomics have been deposited in Mendeley https://data.mendeley.com/datasets/2mc6rrc5j3/2. MS/MS proteomics have been deposited in the PRIDE/ProteomeXchange, http://www.proteomexchange.org/ (accession no. PXD022817). UHPLC-MS metabolomics have been deposited in Metabolomics Workbench, https://www.metabolomicsworkbench.org/ (accession no. PR001110). Mass cytometry data have been deposited in the Flow Repository, https://flowrepository.org/ (accession no. FR-FCM-Z367). Previously published data were used for this work (20, 21).
