# Supplementary material for: Specialized interferon action in COVID-19
Source: Proc Natl Acad Sci U S A. 2022 Feb 25;119(11):e2116730119. doi: 10.1073/pnas.2116730119 (PMC8931386; doi:10.1073/pnas.2116730119)
Supplement: Supplementary File [file pnas.2116730119.sapp.pdf]

PNAS
www.pnas.org

Supplementary Information for
Specialized interferon action in COVID-19

Matthew D. Galbraith[1,2], Kohl T. Kinning[1], Kelly D. Sullivan[1,3], Paula Araya[1], Keith P. Smith[1], Ross E. Granrath[1], Jessica R. Shaw[1], Ryan Baxter[4], Kimberly R. Jordan[4], Seth Russell[5], Monika Dzieciatkowska[6], Julie A. Reisz[6], Fabia Gamboni[6], Francesca Cendali[6], Tusharkanti Ghosh[7], Kejun Guo[8], Cara C. Wilson[8], Mario L. Santiago[8], Andrew A. Monte[9], Tellen D. Bennett[10], Kirk C. Hansen[6], Elena W.Y. Hsieh[4,11], Angelo D'Alessandro[6], and Joaquin M. Espinosa[1,2*]

Affiliations:
[1]Linda Crnic Institute for Down Syndrome, University of Colorado Anschutz Medical Campus; Aurora, CO, USA.
[2]Department of Pharmacology, University of Colorado Anschutz Medical Campus; Aurora, CO, USA. [3]Department of Pediatrics, Section of Developmental Biology, University of Colorado Anschutz Medical Campus; Aurora, CO, USA.
[4]Department of Immunology and Microbiology, University of Colorado Anschutz Medical Campus; Aurora, CO, USA.
[5]Data Science to Patient Value, University of Colorado Anschutz Medical Campus; Aurora, CO, USA.
[6]Department of Biochemistry and Molecular Genetics, University of Colorado Anschutz Medical Campus; Aurora, CO, USA.
[7]Department of Biostatistics and Informatics, Colorado School of Public Health; Aurora, CO, USA.
[8]Department of Medicine, Division of Infectious Diseases, University of Colorado Anschutz Medical Campus; Aurora, CO, USA.
[9]Department of Emergency Medicine, University of Colorado Anschutz Medical Campus; Aurora, CO, USA.
[10]Department of Pediatrics, Sections of Informatics and Data Science and Critical Care Medicine, University of Colorado Anschutz Medical Campus; Aurora, CO, USA.
[11]Department of Pediatrics, Section of Allergy/Immunology, University of Colorado Anschutz Medical Campus; Aurora, CO, USA.

Corresponding author: Joaquin M. Espinosa
Email: joaquin.espinosa@cuanschutz.edu

This PDF file includes:

Extended Methods
Supplementary Figures 1 to 10
Legends for Datasets S1 to S20
SI References

Other supplementary materials for this manuscript include the following:

Supplementary Datasets S1 to S20

EXTENDED METHODS

Data availability.

Data used in this manuscript are available via the COVIDome Explorer online researcher portal (covidome.org), as described in (1). The whole-blood RNA-seq data have been deposited in NCBI Gene Expression Omnibus, with series accession number GSE167000. The PBMC RNA-seq data have been deposited in NCBI Gene Expression Omnibus, with series accession number GSE191317. The SOMAscan® Proteomics, MSD Cytokine Profiles, and Sample Metadata files have been deposited in Mendeley under entry doi:10.17632/2mc6rrc5j3.2. The mass spectrometry proteomics data have been deposited in PRIDE (as part of ProteomeXchange) (2, 3) with the dataset identifier PXD022817. The mass cytometry data has been deposited in Flow Repository (flowrepository.org) (4) with Repository ID FR-FCM-Z367. Raw metabolomics MS data files and annotated reports are available at the NIH Common Fund's National Metabolomics Data Repository (NMDR) website (supported by NIH grant U2C-DK119886), the Metabolomics Workbench (metabolomicsworkbench.org) under the Project ID PR001110, and can be accessed directly via the Project DOI: 10.21228/M8739H.

Blood processing.

Blood samples were collected into EDTA tubes, sodium heparin tubes, and PAXgene Blood RNA Tubes (PreAnalytiX/Qiagen). After centrifugation, EDTA plasma was used for MS proteomics, SOMAscan® proteomics, as well as multiplex immunoassays for both cytokine profiles and seroconversion assays. From sodium heparin tubes, PBMCs were obtained by the Ficoll gradient method before cryopreservation and assembly of batches for MC analysis (see below). All blood samples from the COVID-19-positive cohort were collected when these patients were presenting mild-to-moderate symptomatology. Although some participants were later admitted to the intensive care unit (ICU) or required higher oxygen supplementation, blood draws occurred prior to these events.

Whole-blood RNA library preparation and sequencing.

RNA was purified from PAXgene Blood RNA Tubes (PreAnalytiX/Qiagen) using a PAXgene Blood RNA Kit (Qiagen), according to the manufacturer's instructions. RNA quality was assessed using an Agilent 2200 TapeStation and quantified by Qubit (Life Technologies). Globin RNA depletion, poly-A(+) RNA enrichment, and strand-specific library preparation were carried out using a Universal Plus mRNA-Seq kit with NuQuant, Human Globin AnyDeplete (Tecan). Paired-end 150 bp sequencing was carried out on a NovaSeq 6000 instrument (Illumina) by the Genomics Shared Resource at the University of Colorado Anschutz Medical Campus.

Plasma proteomics by SOMAscan® assays.

125 µL of EDTA plasma was analyzed by SOMAscan® assays using previously established protocols (5). Briefly, each of the 4000+ SOMAmer reagents binds a target peptide and is quantified on a custom Agilent hybridization chip. Normalization and calibration were performed according to SOMAscan® Data Standardization and File Specification Technical Note (SSM-020) (5). The output of the SOMAscan® assay is reported in relative fluorescent units (RFU).

Validation of IFN detection was carried out by spiking recombinant human IFNs into separate aliquots of a pooled plasma reference sample (10 pg/µL). Data were processed as above and then to account for background signal in the reference sample, the median relative abundance measured by each SOMAscan® aptamer reagent across all samples was subtracted from the corresponding values for each spike-in sample. Recombinant human IFNs were obtained from PBL Assay Science (Piscataway, NJ 08854 USA), with the following catalog numbers: 11002-1 (Human Interferon Alpha Sampler Set: IFNA1, IFNA2, IFNA4, IFNA5, IFNA6, IFNA7, IFNA8, IFNA10, IFNA14, IFNA16, IFNA17, IFNA21); 11725-1 (IFNL1); 11720-1 (IFNL2); 11730-1 (IFNL3); 11500-1 (IFNG); 11420-1 (IFNB1).

Cytokine profiling and seroconversion by multiplex immunoassay.

Multiplex immunoassays MSD assays were performed on EDTA plasma aliquots following manufacturer's instructions (Meso Scale Discovery, MSD). *Absolute* values were obtained by extrapolation against a standard curve using provided calibrators.

Validation of IFN detection was carried out by spiking a range of concentrations of recombinant IFNs into separate aliquots of a pooled plasma reference sample followed by measurement as above. Recombinant human IFNs were obtained from PBL Assay Science (Piscataway, NJ 08854 USA), with the following catalog numbers: 11002-1 (Human Interferon Alpha Sampler Set) IFNA2; 11725-1 (IFNL1); 11500-1 (IFNG); 11420-1 (IFNB1).

Seroconversion assays against SARS-CoV-2 proteins were performed in a multiplex immunoassay using the IgG detection readout according to manufacturer's instructions (MSD). *Relative* values were obtained by extrapolation against a standardized curve consisting of pooled COVID-19-positive reference plasma (6).

Plasma proteomics by mass spectrometry.

Plasma samples were digested in S-Trap filters (Protifi, Huntington, NY) according to the manufacturer's procedure. Briefly, a dried protein pellet prepared from organic extraction of patient plasma was solubilized in 400 µl of 5% (w/v) SDS. Samples were reduced with 10 mM DTT at 55°C for 30 min, cooled to room temperature, and then alkylated with 25 mM iodoacetamide in the dark for 30 min. Next, a final concentration of 1.2% phosphoric acid and then six volumes of binding buffer [90% methanol; 100 mM triethylammonium bicarbonate (TEAB); pH 7.1] were added to each sample. After gentle mixing, the protein solution was loaded into an S-Trap filter, spun at 2000 rpm for 1 min, and the flow-through collected and reloaded onto the filter. This step was repeated three times, and then the filter was washed three times with 200 µL of binding buffer. Finally, 1 µg of sequencing-grade trypsin (Promega) and 150 µL of digestion buffer (50 mM TEAB) were added onto the filter and digestion carried out at 47 °C for 1 h. To elute peptides, three stepwise buffers were applied, 200 µL of each with

one more repeat, including 50 mM TEAB, 0.2% formic acid (FA) in H_2O, and 50% acetonitrile and 0.2% formic acid in H_2O. The peptide solutions were pooled, lyophilized and resuspended in 1 mL of 0.1 % FA. 20 µl of each sample was loaded onto individual Evotips for desalting and then washed with 20 µL 0.1% FA followed by the addition of 100 µL storage solvent (0.1% FA) to keep the Evotips wet until analysis. The Evosep One system (Evosep, Odense, Denmark) was used to separate peptides on a Pepsep column (150 µm internal diameter, 15 cm) packed with ReproSil C18 1.9 µm, 120A resin. The system was coupled to a timsTOF Pro mass spectrometer (Bruker Daltonics, Bremen, Germany) via a nano-electrospray ion source (Captive Spray, Bruker Daltonics). The mass spectrometer was operated in PASEF mode. The ramp time was set to 100 ms and 10 PASEF MS/MS scans per topN acquisition cycle were acquired. MS and MS/MS spectra were recorded from m/z 100 to 1700. The ion mobility was scanned from 0.7 to 1.50 Vs/cm^2. Precursors for data-dependent acquisition were isolated within ± 1 Th and fragmented with an ion mobility-dependent collision energy, which was linearly increased from 20 to 59 eV in positive mode. Low-abundance precursor ions with an intensity above a threshold of 500 counts but below a target value of 20000 counts were repeatedly scheduled and otherwise dynamically excluded for 0.4 min. Raw data file conversion to peak lists in the MGF format, downstream identification, validation, filtering and quantification were managed using FragPipe version 13.0. MSFragger version 3.0 was used for database searches against a Human isoform-containing UniProt fasta file (version 08/11/2020) with decoys and common contaminants added. The identification settings were as follows: Trypsin, Specific, with a maximum of 2 missed cleavages, up to 2 isotope errors in precursor selection allowed for, 10.0 ppm as MS1 and 20.0 ppm as MS2 tolerances; fixed modifications: Carbamidomethylation of C (+57.021464 Da), variable modifications: Oxidation of M (+15.994915 Da), Acetylation of protein N-term (+42.010565 Da), Pyrolidone from peptide N-term Q or C (-17.026549 Da). The Philosopher toolkit version 3.2.9 (build 1593192429) was used for filtering of results at the peptide and protein level at 0.01 FDR. Label-free quantification was performed by AUC integration with matching between all runs using IonQuant.

Mass cytometry analysis of immune cell types.

Cryopreserved PBMCs were thawed, washed twice with Cell Staining Buffer (CSB) (Fluidigm), and counted with an automated cell counter (Countess II, Thermo Fisher Scientific). Extracellular staining on live cells was done in CSB for 30 min at room temperature, in 3-5 x 10^6 cells per sample. Cells were washed with 1X PBS (Fluidigm) and stained with 1 mL of 0.25 mM cisplatin (Fluidigm) for 1 min at room temperature for exclusion of dead cells. Samples were then washed with CSB and incubated with 1.6% PFA (Electron Microscopy Sciences) for 10 min at room temperature. Samples were washed with CSB and barcoded using a Cell-IDTM 20- Plex Pd Barcoding Kit (Fluidigm) of lanthanide-tagged cell reactive metal chelators to covalently label samples with a unique combination of palladium isotopes, then combined. Surface staining with antibodies that work on fixed epitopes was performed in CSB for 30 min at room temperature (see **Supplementary Data 15** for antibody information). Cells were washed twice with CSB and fixed in Fix/Perm buffer (eBioscience) for 30 min, washed twice in permeabilization buffer (eBioscience), then intracellular factors were stained in permeabilization buffer for 45 min at 4°C. Cells were washed twice with Fix/Perm Buffer and labeled overnight at 4°C with Cell-ID Intercalator-Ir (Fluidigm) for DNA staining. Cells were then analyzed on a Helios instrument (Fluidigm). To make all samples comparable, pre-processing of mass cytometry data included normalization within and between batches via polystyrene beads embedded with lanthanides as previously described (7). Files were debarcoded using the Matlab DebarcoderTool (8). Finally, normalization was carried out between batches relative to a reference batch based on technical replicates (9).

Mass spectrometry-based metabolomics of plasma and red blood cells.

Sample extraction. Samples were thawed on ice and extracted via a modified Folch method (chloroform/methanol/water 8:4:3), which completely inactivates other coronaviruses, such as MERS-CoV. Briefly, 20 µL of sample was diluted in 130 µL of LC-MS grade water, 600 µL of ice-cold chloroform/methanol (2:1) was added, and the samples were vortexed for 10 seconds. Samples were then incubated at 4°C for 5 minutes, quickly vortexed (5 seconds), and centrifuged at 14,000 *g* for 10

minutes at 4°C. The top (i.e., aqueous) phase was transferred to a new tube for metabolomics analysis and flash frozen. The bottom (i.e., organic) phase was transferred to a new tube for lipidomics analysis, then dried under N_2 flow.

UHPLC-MS metabolomics. Analyses were performed using a Vanquish UHPLC coupled online to a Q Exactive high resolution mass spectrometer (Thermo Fisher Scientific, Bremen, Germany). Samples (10 uL per injection) were randomized and analyzed in positive and negative electrospray ionization modes (separate runs) using a 5-minute C18 gradient on a Kinetex C18 column (Phenomenex) as described (10). Data were analyzed using Maven (Princeton University, Princeton, NJ, USA) in conjunction with the KEGG database and an in-house standard library.

Analysis of whole-blood transcriptome data.

RNA-seq data yield was ~40-80 x 10^6 raw reads and ~32-71 x 10^6 final mapped reads per sample. Reads were demultiplexed and converted to fastq format using bcl2fastq (bcl2fastq v2.20.0.422). Data quality was assessed using FASTQC (v0.11.5) (https://www.bioinformatics.babraham.ac.uk/projects/fastqc/) and FastQ Screen (v0.11.0, https://www.bioinformatics.babraham.ac.uk/projects/fastq_screen/). Trimming and filtering of low-quality reads was performed using bbduk from BBTools (v37.99)(11) and fastq-mcf from ea-utils (v1.05, https://expressionanalysis.github.io/ea-utils/). Alignment to the human reference genome (GRCh38) was carried out using HISAT2 (v2.1.0)(12) in paired, spliced-alignment mode with a GRCh38 index and a Gencode v33 annotation GTF, and alignments were sorted and filtered for mapping quality (MAPQ > 10) using Samtools (v1.5)(13). Gene-level count data were quantified using HTSeq-count (v0.6.1)(14) with the following options (--stranded=reverse –minaqual=10 –type=exon --mode=intersection-nonempty) using a Gencode v33 GTF annotation file. Differential gene expression in COVID+ versus COVID- was evaluated using DESeq2 (version 1.28.1)(15) in R (version 4.0.1), q < 0.1 (FDR < 10%) as the threshold for differentially expressed genes.

Analysis of SOMAscan® data.

Normalized data (RFU) was imported and converted from a SOMAscan® .adat file using a custom R package (SomaDataIO v3.1.0, https://github.com/SomaLogic/SomaDataIO) for use in all subsequent analysis.

Interferon stimulation of *ex vivo* PBMCs.

Peripheral blood mononuclear cells (PBMCs, n=7 anonymous uninfected donors) were procured from Zen-Bio and 1 x 10^6 cells were treated with mock (phosphate-buffered saline), 0.504 nM IFNα2, or 0.070 nM IFNβ (PBL Assay Science). These concentrations were normalized to give equivalent IFN-stimulated response element (ISRE)-luciferase activity in iLite cells (Svar Life Science AB), as described previously (16). After 18 h stimulation, total RNA was extracted using an RNeasy Mini Kit (Qiagen) and libraries constructed using 500 ng of RNA using the QuantSeq 3' mRNAseq (Forward) library kit (Lexogen). Library integrity was evaluated using a 2100 BioAnalyzer (Agilent) prior to paired-end 150 bp sequencing on a NovaSeq 6000 instrument (Illumina) by the Genomics Shared Resource at the University of Colorado Anschutz Medical Campus.

Analysis of PBMC transcriptome data.

RNA-seq data yield was ~27.4-100.8 x 10^6 raw reads and ~7.8-23.3 x 10^6 final mapped reads per sample. As recommended by the library kit manufacturer (Lexogen), only read 1 was retained for analysis. Data quality was assessed using FASTQC. Adapter removal per-base quality screening and trimming of the first 12 bases were performed with cutadapt (https://cutadapt.readthedocs.io/en/stable). Alignment to the human reference genome (GRCh38.p12, Gencode) was carried out using using Hisat2 (12). Gene-level counts were obtained using *featureCounts* from the Subread package (17) and normalized using the trimmed mean of M-values method. Lowly-expressed genes with average read counts less than 5 per library were removed. Differential gene expression in interferon-stimulated

versus mock-treated samples was evaluated using edgeR (18) in R (v4.0.2), with q < 0.1 (FDR < 10%) as the threshold for differentially expressed genes.

Analysis of MSD cytokine profiling data.

Plasma concentration values (pg/mL) for each of the cytokines and related immune factors measured across multiple MSD assay plates was imported to R, combined, and analytes with >10% of values outside of detection or fit curve range flagged. For each analyte, missing values were replaced with either the minimum (if below fit curve range) or maximum (if above fit curve range) calculated concentration and means of duplicate wells used in all further analysis.

Analysis of MS-proteomics data.

Raw Razor intensity data were filtered for high abundance proteins by removing those with >70% zero values in both COVID-19-negative and COVID-19-positive groups. For the remaining 407 abundant proteins, zero values (8,363 missing values of 44,363 total measurements) were replaced with a random value sampled from between 0 and 0.5x the minimum non-zero intensity value for that protein. Data was then normalized using a scaling factor derived from the global median intensity value across all proteins / sample median intensity across all proteins (19).

Analysis of mass cytometry data.

MC data was exported as individual FCS files for each sample. Within the cytofkit package graphical user interface (v1.11.3) (20), FCS files were imported to R (v4.0.3) using the read.FCS() function from the flowCore package (v2.2.0) (21), raw intensity values inverse hyperbolic sine transformed using the cytofAsinh() function with cofactor = 5 from the cytofkit package, and 1000 cells per FCS file sampled without replacement for downstream analysis. For visualization, dimensionality reduction was performed using the t-distributed stochastic neighbor embedding (t-SNE) method from the Rtsne package (v0.15) (22), using all markers. Unsupervised clustering, using all markers, was performed

using the cytofkit implementation of the PhenoGraph algorithm (23). Transformed marker expression values for each clustered cell/event were exported and Z-scores calculated across all events for visualization on t-SNE plots. Relative frequencies for each cluster were calculated as proportions of live cells per sample for use in subsequent analyses. For traditionally gated cell subpopulations (gating strategy is described in (24)), relative frequencies were exported from CellEngine as percentages of various parental lineages for use in subsequent analyses.

Analysis of LCMS-metabolomics data.

Peak intensity data was imported to R. Across the 171 metabolites, 0 values (486 missing values of 21,033 total measurements) were replaced with a random value sampled from between 0 and 0.5x the minimum non-zero intensity value for that metabolite. For downstream analysis, data was then normalized using a scaling factor derived by dividing the global median intensity value across all proteins by each sample median intensity. Median normalization was chosen as it is simple to employ, relies on few assumptions, and performs on-par with more complex normalization techniques, such as linear regression, local regression, total intensity, average intensity, and quantile normalization, in reducing intragroup variation (25), and is one of the non-reference-based normalization methods employed in the widely-used MetaboAnalyst pre-processing module (26).

Gene Set Enrichment Analysis (GSEA).

GSEA (27) was carried out using the fgsea package (v 1.14.0) (28) in R (version 4.0.1), using Hallmark gene sets (29) and either $\log_2$-transformed fold-changes (for RNA-seq and SOMAscan®) or Spearman *rho* values (for IFN correlations) as the ranking metric.

Interferon Alpha/Gamma Scores.

To capture interferon signaling in each sample as a single value we calculated RNA-seq- or SOMAscan®-based 'Interferon Alpha' and 'Interferon Gamma' scores as follows: Firstly, Z-scores were

calculated from the age- and sex-adjusted concentration values for each gene/protein in each sample, based on the mean and standard deviation of COVID-19-negative samples. Secondly, per-sample scores were calculated as the sum of Z-scores for genes/proteins in the Hallmark Interferon Alpha or Hallmark Interferon Gamma Response gene sets (29), filtered to genes/proteins with significant increases in the COVID-19-positive group (see next section): RNA-based IFN Alpha score, 51 genes; RNA-based IFN Gamma score, 84 genes; protein-based IFN Alpha score, 14 proteins; protein-based IFN Gamma score, 23 proteins.

Differential abundance analysis.

For RNAseq, gene-level differential expression in COVID-19+ versus COVID-19- samples was evaluated using DESeq2 (version 1.28.1)(15) in R (version 4.0.1), with $q < 0.1$ (FDR < 10%) as the threshold for differentially expressed genes, and considering only genes with ≥ 0.5 counts-per-million in at least two samples. Differential abundance analysis for SOMAscan® proteomics (Figure 2) was performed using linear regression in R (version 4.0.1) with $\log_2$ aptamer abundance as the outcome/dependent variable and COVID-19 status as the predictor/independent variable, with adjustment for Age and Sex. Extreme outlier data points (above Q3 + 3*IQR or below Q1 – 3*IQR) were removed. Multiple hypothesis correction was performed with the Benjamini-Hochberg method using a false discovery rate (FDR) threshold of 10% ($q < 0.1$). Differential abundance analysis for IFNs against clinical demographic features (Figure 6), was performed using linear regression in R (version 4.0.1) with $\log_2$ interferon concentration/abundance as the outcome/dependent variable and age (continuous), sex (males/females), ICU status (ever ICU/never ICU), O_2 group (High/Low), or days since admission (continuous, limited to ≤14 days) as the predictor/independent variables, with adjustment for age and/or sex as appropriate, for COVID-19-positive samples only. Extreme outlier data points (above Q3 + 3*IQR or below Q1 – 3*IQR) were removed. Multiple hypothesis correction was performed with the Benjamini-Hochberg method using a false discovery rate (FDR) threshold of 10% ($q < 0.1$).

Beta regression analysis of MC data.

To identify cell clusters or gated cell subsets for which relative frequencies are associated with plasma levels of the 12 IFNs in COVID-19-positive samples, beta regression analysis was carried out using the betareg package (v3.1-4) (30), with each model using cell cluster/subset proportions (relative frequency) as the outcome/dependent variable and log2-transformed IFN abundance values as the independent/predictor variable, with adjustment for Age and Sex, and a logit link function. Effect sizes (as fold-change per unit IFN abundance) for each IFN were obtained by exponentiation of beta regression model coefficients. For comparison across IFNs as in volcano plots and heatmaps, beta regression model coefficients were multiplied by the standard deviation of the corresponding IFN before exponentiating to give 'standardized' fold-changes per standard deviation of IFN abundance. Standardized fold-changes from each model were visualized by overlaying on t-SNE plots or as heatmaps using the ggplot2 (v3.3.1) (31) and ComplexHeatmap (v2.4.2) (32) packages. For visualization of individual IFN vs. cluster/subset examples, data points were visualized as XY scatter plots, with points colored by local density using a custom function, and overlaid with beta regression fit curves and 95% confidence intervals extracted from model objects using the *ggemmeans()* function from the ggeffects package (v1.1.0) (33).

Correlation analysis.

To identify features in each dataset that correlate with plasma levels of the 12 IFNs in COVID-19-positive samples, Spearman *rho* values and p-values were calculated against the Sex/Age-adjusted values for each dataset using the *rcorr* function from the Hmisc package (v 4.4-0) (34), with Benjamini-Hochberg correction of p-values and an estimated FDR threshold of 0.1. Extreme outlier data points (above Q3 + 3*IQR or below Q1 – 3*IQR) were removed. For visualization, Heatmaps and XY scatter plots, with points colored by local density using a custom density function, were generated using the ComplexHeatmap (v2.4.2) (32) and the ggplot2 (v3.3.1) (31) packages.

Supplementary Figure 1

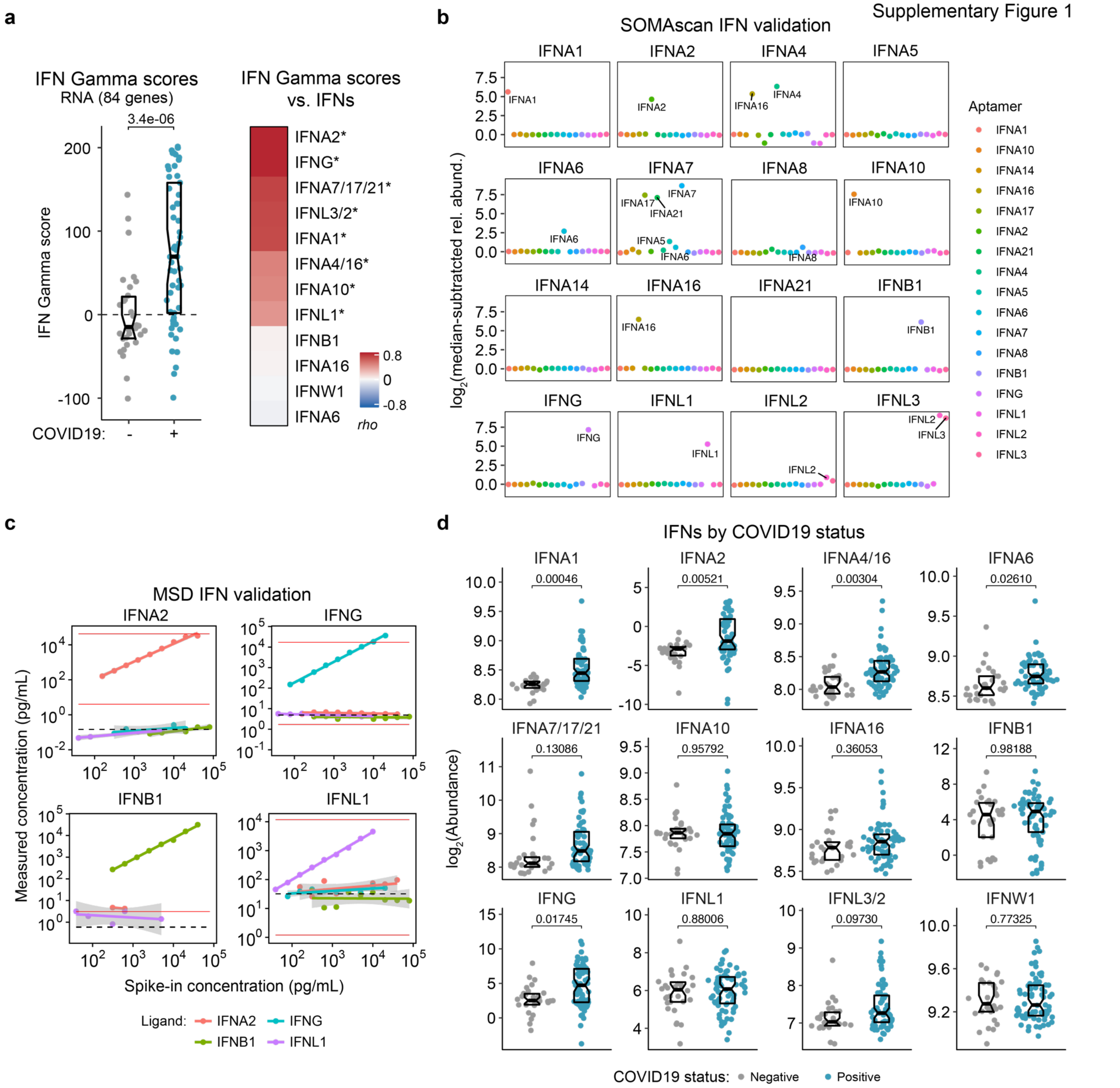

Supplementary Fig. 1. Whole-blood RNA-based IFN Gamma scores and validation of plasma IFNs measurements. (**a**) Sina plot of RNA-based IFN Gamma scores, separated by COVID-19 status and ranked heatmap representing correlations between RNA-based IFN Gamma scores and plasma levels of each IFN. Data are presented as a modified sina plot with box indicating median and interquartile range. Heatmap values displayed are Spearman correlation coefficients (Rho); asterisks indicate significant correlations (10% FDR). (**b**) Validation of IFN detection by SOMAscan® assay. Each plot represents relative abundance above background measured by IFN-targeting SOMAscan® aptamers (indicated by color) for each recombinant IFN spike-in (indicated by plot labels). (**c**) Validation of IFN detection by MSD immunoassay. Plots show the relationship between measured concentration and spike-in concentration for each recombinant IFN (indicated by point and line color) for each assay (indicated by plot labels). Horizontal dashed lines indicate measured concentrations for the pooled plasma sample with no spike-in; red lines indicate manufacturer-stated detection limits. (**d**) Sina plots comparing abundance for the indicated IFNs in COVID-19-negative (-) vs. -positive (+) plasma samples. Data are presented as modified sina plots with boxes indicating median and interquartile range. Numbers above brackets are q-values for Mann–Whitney tests.

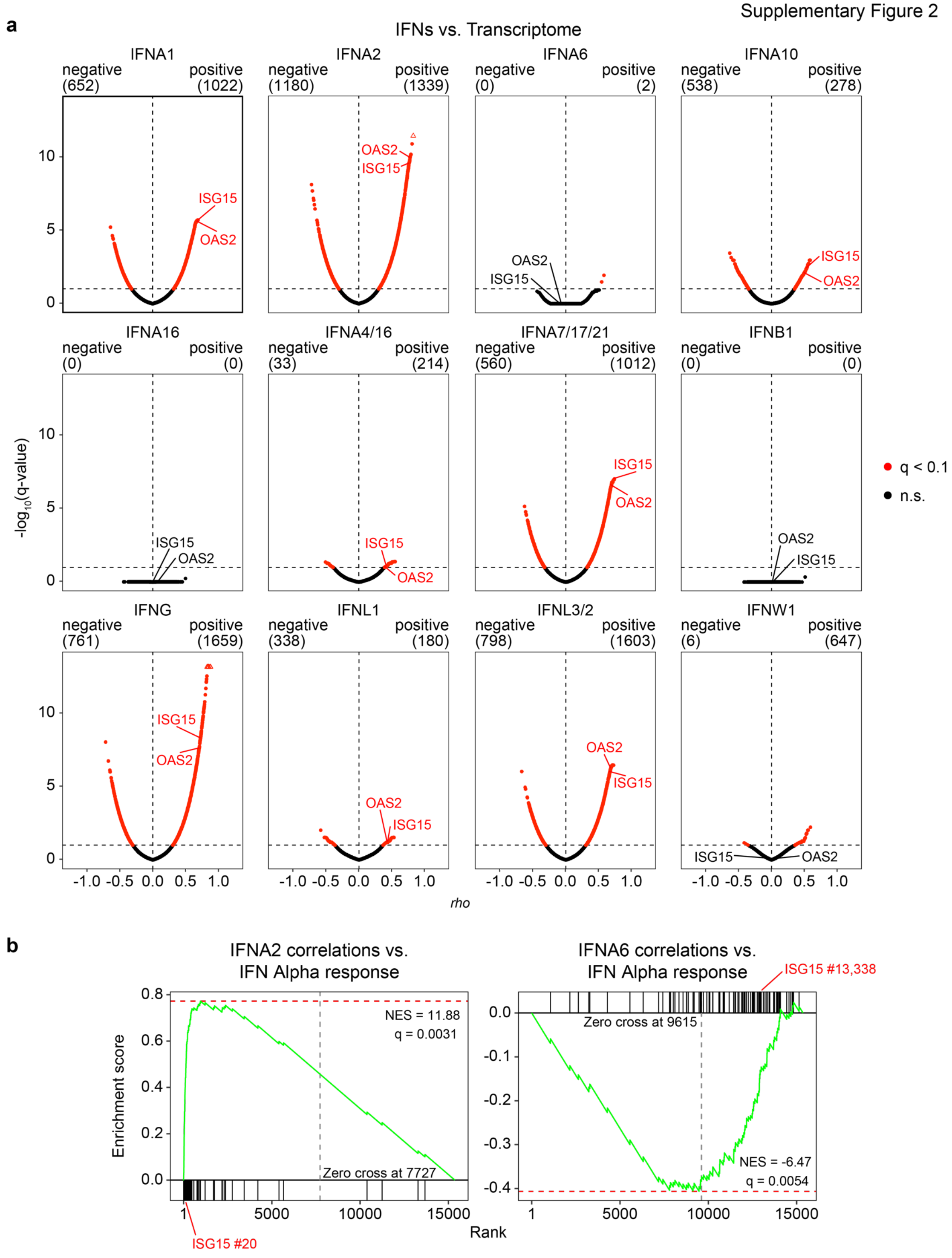

Supplementary Fig. 2. Correlation analysis and GSEA for IFNs vs. whole blood transcriptome. (**a**) Volcano plots for Spearman correlation analysis of IFNs vs. gene-level RPKM values. Horizontal dashed line indicates an FDR threshold of 10% (q < 0.1); red points and numbers above plots indicate significant genes at this threshold. (**b**) Gene set enrichment analysis (GSEA) plots for the IFN Alpha Response Hallmark gene set from MSigDB. Green lines indicate cumulative enrichment score; black bars indicate gene set hits among all genes ranked by log2(fold change) for COVID-19-positive vs. -negative samples.

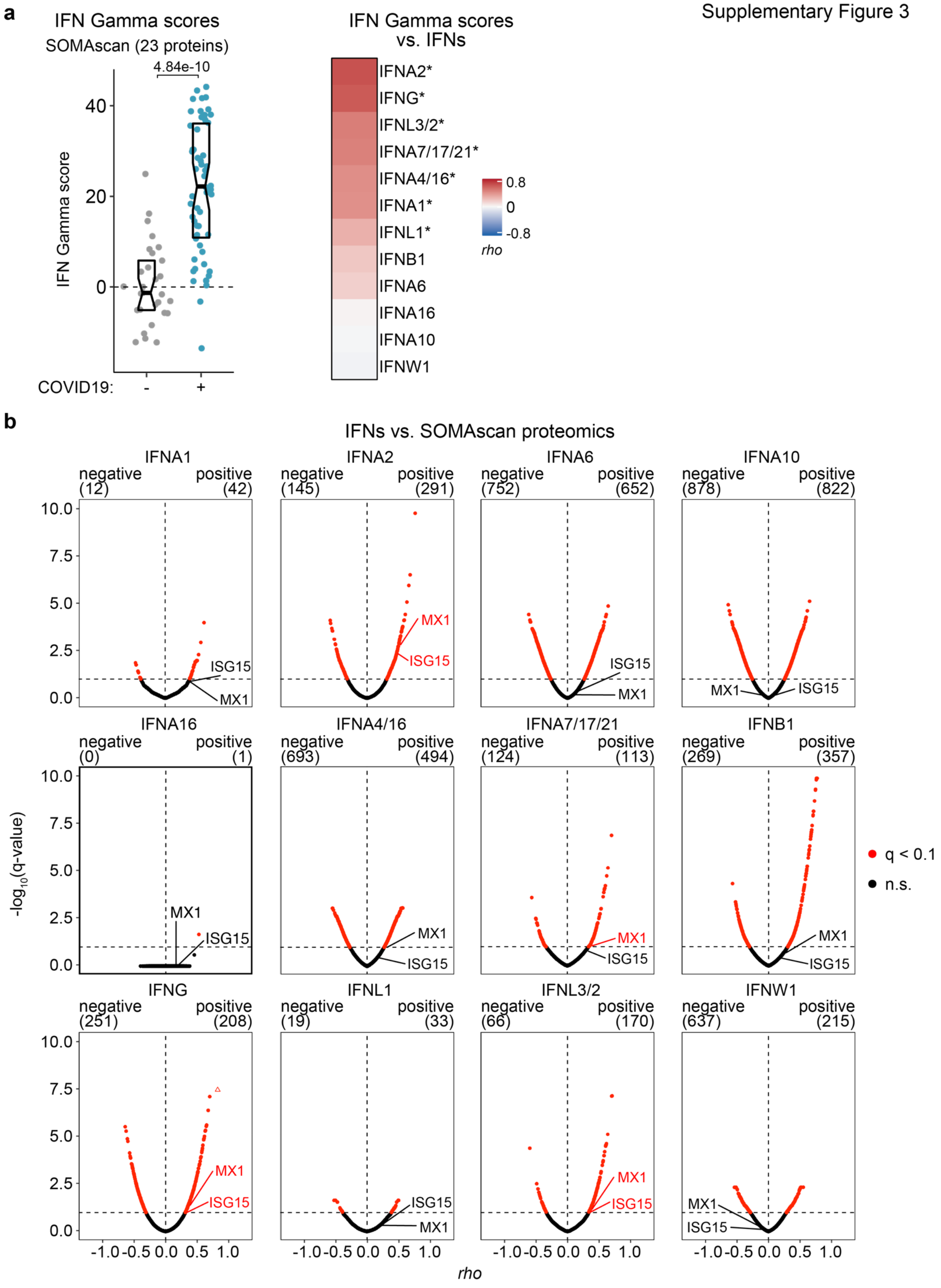

Supplementary Fig. 3. Plasma protein-based IFN Gamma scores and correlation analysis for IFNs vs. SOMAscan® proteomics. (**a**) Sina plot of protein-based IFN Gamma scores, separated by COVID-19 status and ranked heatmap representing correlations between protein-based IFN Gamma scores and plasma levels of each IFN. Data are presented as a modified sina plot with box indicating median and interquartile range. Heatmap values displayed are Spearman correlation coefficients (Rho); asterisks indicate significant correlations (10% FDR). (**b**) Volcano plots for Spearman correlation analysis of IFNs vs. SOMAscan® protein abundance values. Horizontal dashed line indicates an FDR threshold of 10% (q < 0.1); red points and numbers above plots indicate significant proteins at this threshold.

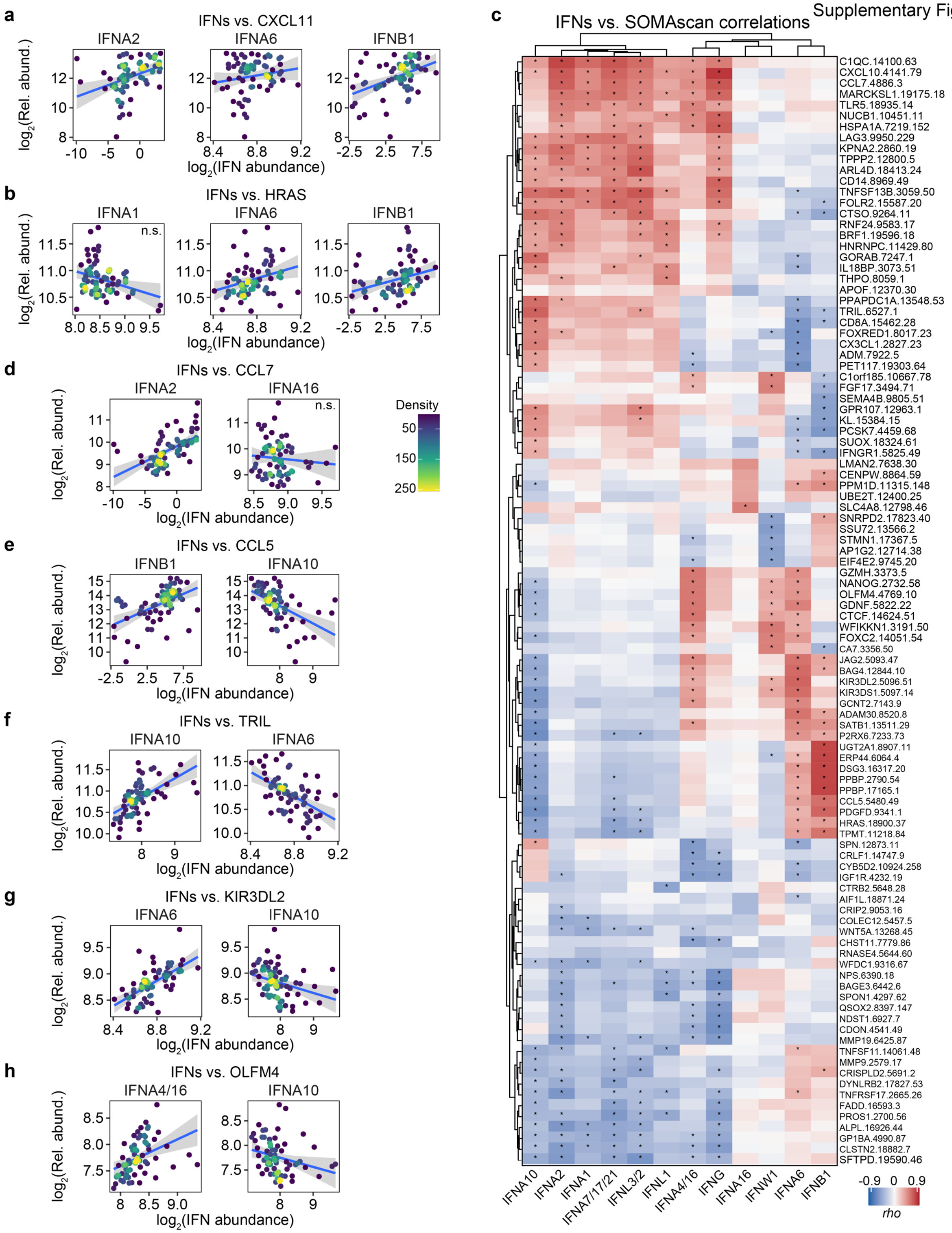

Supplementary Fig. 4. Relationships between IFNs and SOMAscan® plasma proteomics. (**a-b** and **d-h**) Scatter plots comparing relationships between plasma proteins and the indicated IFNs in COVID-19-positive patients. Points are colored by density; blue lines represent linear model fit with 95% confidence intervals in grey. (**c**) Heatmap representing correlations between plasma levels of proteins measured by SOMAscan® and each IFN. Values displayed are Spearman correlation scores (Rho) for

proteins ranked in top 5 positive or top 5 negative correlations for at least one IFN; asterisks indicate significant correlations (10% FDR); columns and rows are grouped by hierarchical clustering.

Supplementary Figure 5

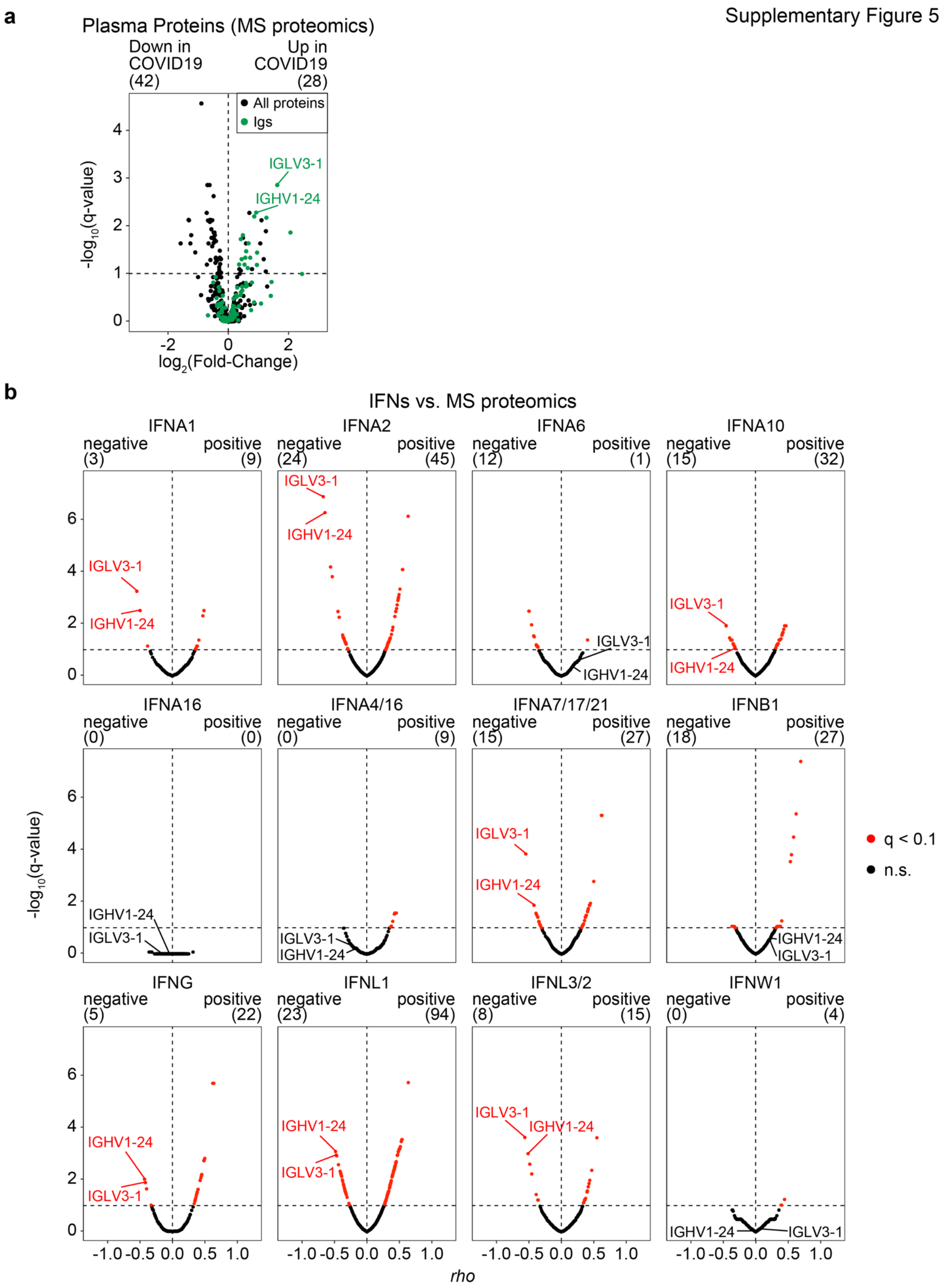

Supplementary Fig. 5. Differential abundance and correlation analysis for MS plasma proteomics. (**a**) Volcano plot for linear regression analysis of MS proteomics plasma protein abundance data for COVID-19-positive vs. -negative samples, adjusted for age and sex. Horizontal dashed line indicates an FDR threshold of 10% ($q < 0.1$); numbers above plot indicate significant genes at this threshold. Immunoglobulin subunits (IGs) are highlighted in green. (**b**) Volcano plots for Spearman correlation analysis of IFNs vs. MS proteomics protein relative abundance values. Horizontal dashed line indicates an FDR threshold of 10% ($q < 0.1$); red points and numbers above plots indicate significant proteins at this threshold.

Supplementary Figure 6

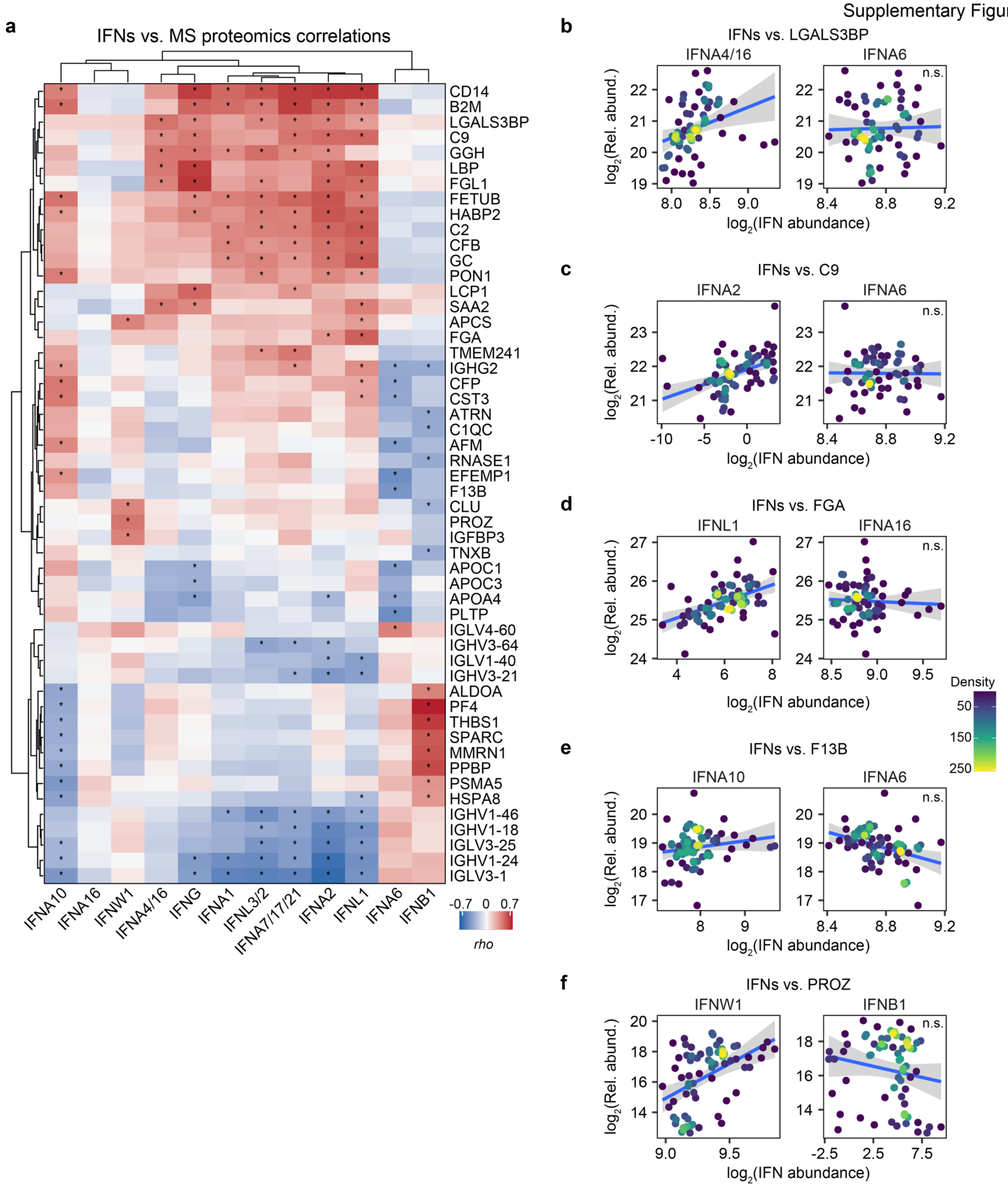

Supplementary Fig. 6. Relationships between IFNs and MS plasma proteomics. (**a**) Heatmap representing correlations between IFNs and plasma protein levels, as measured by MS proteomics. Values displayed are Spearman correlation scores (Rho) for proteins ranked in top 5 positive or top 5 negative correlations for at least one IFN; asterisks indicate significant correlations (10% FDR); columns and rows are grouped by hierarchical clustering. (**b-f**) Scatter plots comparing relationships between plasma proteins, as measured by MS proteomics, and the indicated IFNs in COVID-19-positive patients. Points are colored by density; blue lines represent linear model fit with 95% confidence intervals in grey.

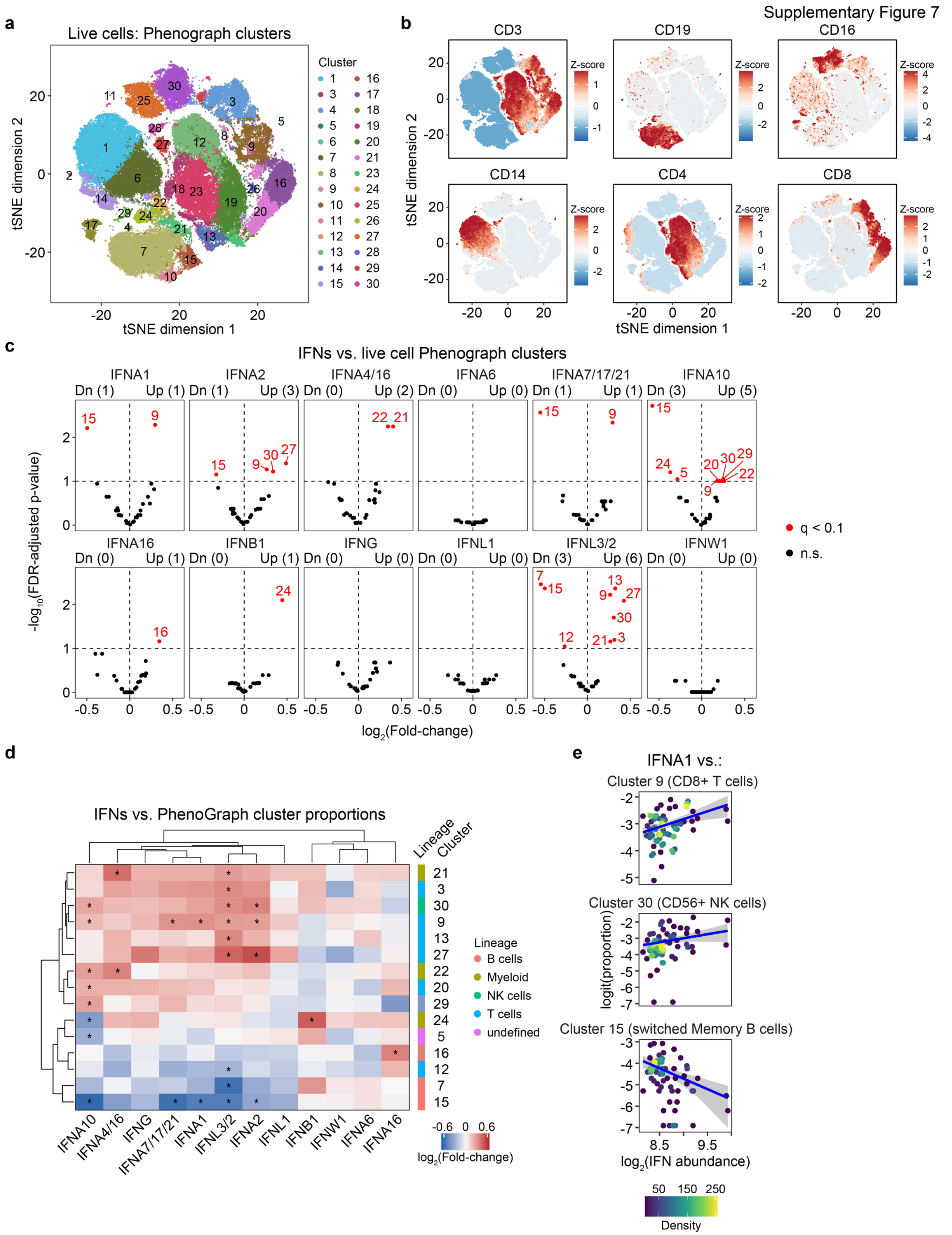

Supplementary Fig. 7. PhenoGraph clustering and beta regression analysis of clustered mass cytometry data against IFNs. (**a**) t-SNE plots of 69,000 cells analyzed by mass cytometry from 69 COVID-19-positive patients (1,000 cells each). Numbers and coloring of cells indicate PhenoGraph cluster assignments. (**b**) t-SNE plots with cells colored by Z-scores for markers of T cells (CD3, CD4,

CD8), B cells (CD19), NK cells (CD16), and Monocytes (CD14). (**c**) Volcano plots for Beta regression analysis of IFNs vs. cluster proportions among live cells, adjusted for age and sex. X-axes display log2-transformed fold-change in cluster proportion among live cells per standard deviation of IFN abundance; horizontal dashed line indicates an FDR threshold of 10% ($q < 0.1$); red points and numbers above plots indicate significant clusters at this threshold. (**d**) Heatmap representing relationships between IFNs and cluster proportions among live cells, as determined by beta regression analysis. Only subpopulations with at least one significant association are shown. Values displayed are fold-change in cluster proportion among live cells per standard deviation of IFN abundance; asterisks indicate significant associations (10% FDR); columns and rows are grouped by hierarchical clustering. (**e**) Scatter plots comparing relationships between cluster proportions among live cells, and the indicated IFNs in COVID-19-positive patients. Points are colored by density; blue lines represent beta regression model fit with 95% confidence intervals in grey.

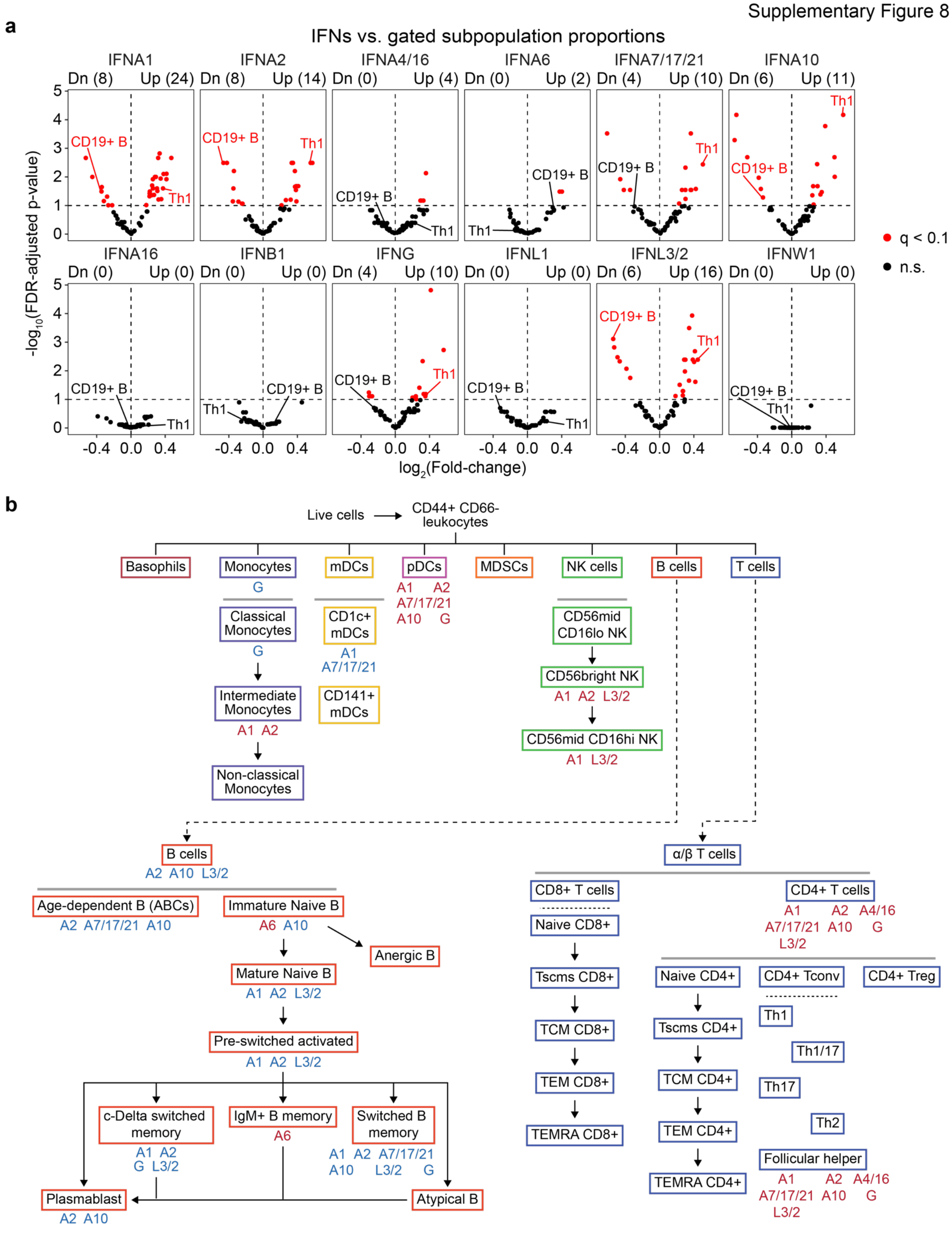

Supplementary Fig. 8. Beta regression analysis of gated mass cytometry data against IFNs. (**a**) Volcano plots for Beta regression analysis of IFNs vs. gated subpopulation proportions among live cells, adjusted for age and sex. X-axes display log2-transformed fold-change in cluster proportion among live cells per standard deviation of IFN abundance; horizontal dashed line indicates an FDR threshold of 10% (q < 0.1); red points and numbers above plots indicate significant subpopulations at this threshold. (**b**) Cell lineage map indicating the relationships between gated cell subpopulations included in beta regression analysis. Boxed labels represent subpopulations for which relative cell frequencies were obtained. Grey horizontal lines denote subpopulations of cells derived by gating; black lines and arrows indicate subpopulations that are also related by cell differentiation; labels outside boxes indicate significant positive (red) and negative (blue) relationships with IFNs.

Supplementary Figure 9

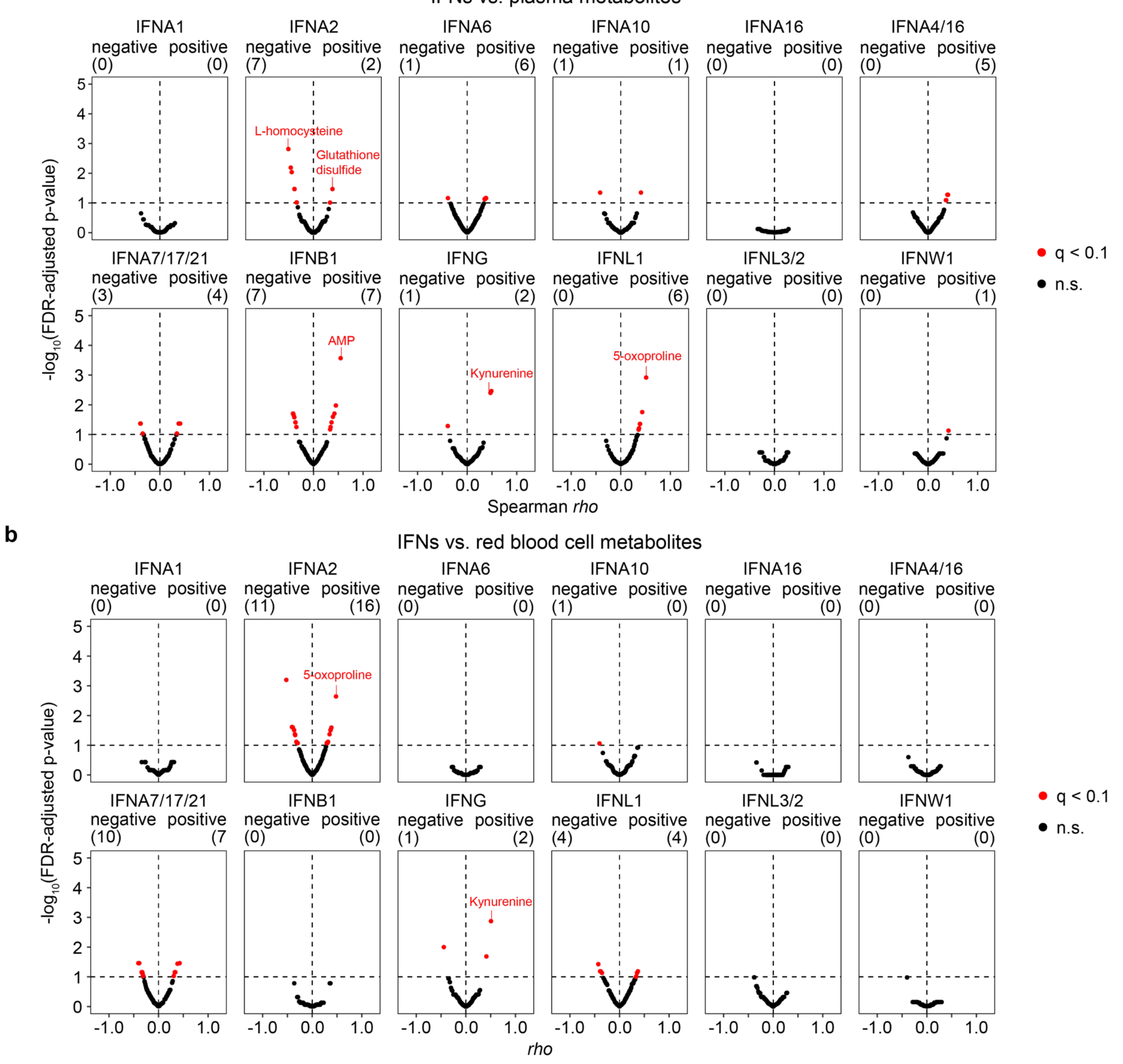

Supplementary Fig. 9. Correlation analysis of plasma and RBC metabolites vs. IFNs. (**a-b**) Volcano plots for Spearman correlation analysis of IFNs vs. plasma (a) or RBC (b) metabolite relative abundance values. Horizontal dashed line indicates an FDR threshold of 10% (q < 0.1); red points and numbers above plots indicate significant metabolites at this threshold.

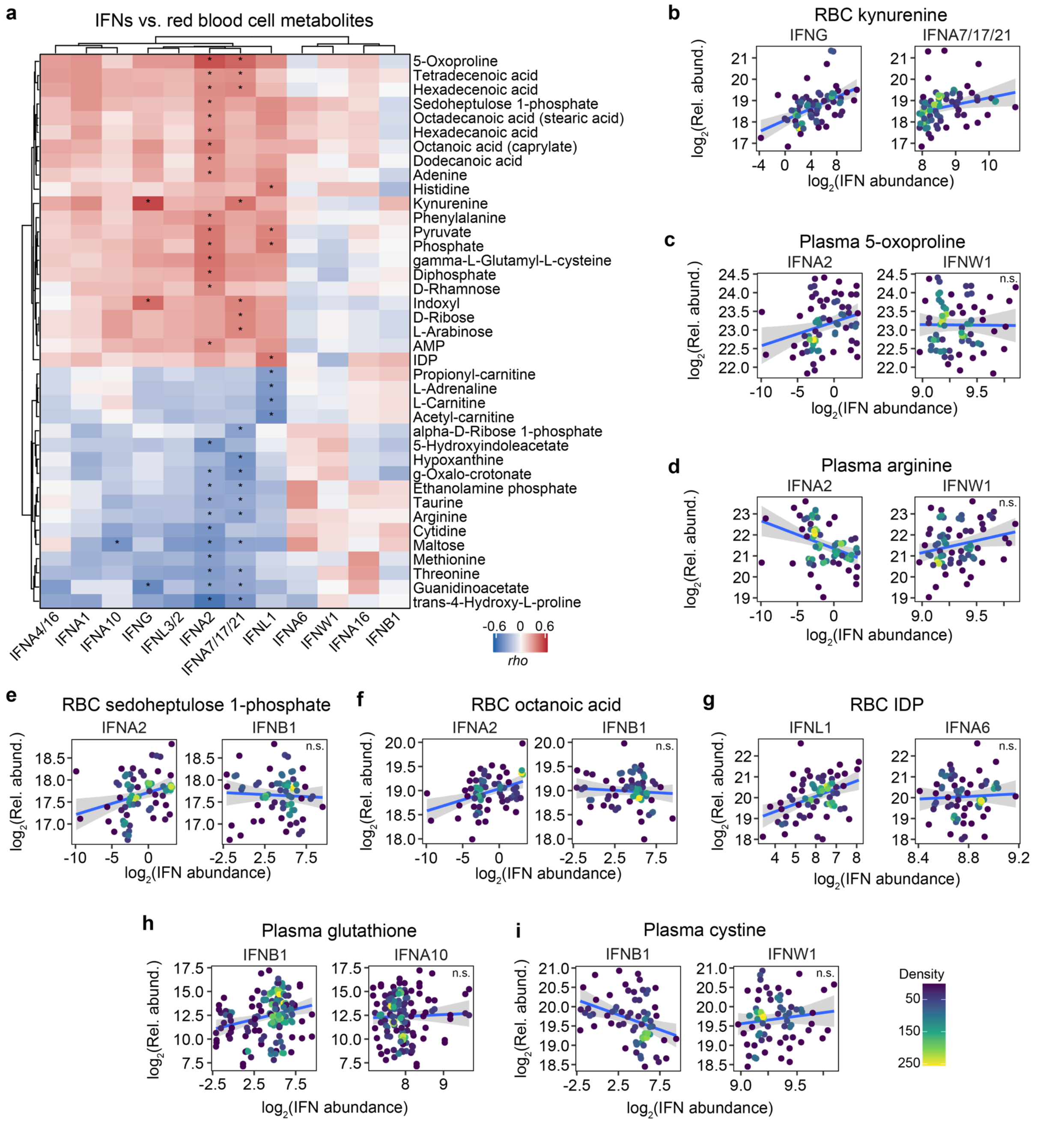

Supplementary Fig. 10. Correlation analysis of plasma and RBC metabolites vs. IFNs. (**a**). Heatmap representing correlations between IFNs and red blood cell (RBC) metabolite levels. Only metabolites with at least one significant correlation are shown. Values displayed are Spearman correlation scores (*Rho*); asterisks indicate significant correlations (10% FDR); columns and rows are grouped by hierarchical clustering. (**b-i**) Scatter plots comparing relationships between select metabolites and the indicated IFNs in COVID-19-positive patients. Points are colored by density; blue lines represent linear model fit with 95% confidence intervals in grey.

SUPPLEMENTARY DATA FILES

Supplementary Data 1 (separate file). Cohort characteristics. Table summarizing cohort characteristics. Cells with totals less than 10 are indicated as <10 to prevent potential reidentification of these participants.

Supplementary Data 2 (separate file). Whole-blood transcriptome differential expression by COVID status. Results of DESeq2 differential expression analysis of whole-blood RNA-seq data from COVID-19-positive vs. -negative samples.

Supplementary Data 3 (separate file). GSEA of transcriptome by COVID status. Results from Gene Set Enrichment Analysis (GSEA) of Hallmark gene sets using RNA-seq fold-change COVID-19-positive vs. negative as the ranking metric.

Supplementary Data 4 (separate file). IFNs vs transcriptome correlations. Results of Spearman correlation analysis between plasma levels of IFNs and whole blood RNA-seq gene-level expression in COVID-19-positive samples.

Supplementary Data 5 (separate file). GSEA of IFNs vs transcriptome correlations. Results from Gene Set Enrichment Analysis (GSEA) of Hallmark gene sets using Spearman correlation scores for IFNs vs. whole blood RNA-seq gene-level expression as the ranking metric.

Supplementary Data 6 (separate file). SOMAscan® proteomics differential abundance by COVID-19 status. Results of linear model differential abundance analysis of plasma SOMAscan® proteomics data in COVID-19-positive vs. -negative samples.

Supplementary Data 7 (separate file). GSEA of SOMAscan® proteomics by COVID status. Results from Gene Set Enrichment Analysis (GSEA) of Hallmark gene sets using SOMAscan® proteomics fold-change COVID-19-positive vs. negative as the ranking metric.

Supplementary Data 8 (separate file). IFNs vs SOMAscan® proteomics correlations. Results of Spearman correlation analysis between plasma levels of IFNs and SOMAscan® proteomics data in COVID-19-positive samples.

Supplementary Data 9 (separate file). GSEA of IFNs vs SOMAscan® proteomics correlations. Results from Gene Set Enrichment Analysis (GSEA) of Hallmark gene sets using Spearman correlation scores for IFNs vs. SOMAscan® proteomics data as the ranking metric.

Supplementary Data 10 (separate file). PBMC transcriptome differential expression by IFNA2 versus IFNB1 stimulation. Results of EdgeR differential expression analysis RNA-seq data from *ex vivo* PBMCs.

Supplementary Data 11 (separate file). MS proteomics differential abundance by COVID status. Results of linear model differential abundance analysis of plasma mass spectrometry (MS) proteomics data in COVID-19-positive vs. -negative samples.

Supplementary Data 12 (separate file). IFNs vs MS proteomics correlations. Results of Spearman correlation analysis between plasma levels of IFNs and mass spectrometry (MS) proteomics data in COVID-19-positive samples.

Supplementary Data 13 (separate file). IFNs vs mass cytometry clusters beta regression. Results of beta regression analysis of relative frequency data for PhenoGraph-defined subpopulation clusters against plasma levels of IFNs in COVID-19-positive samples.

Supplementary Data 14 (separate file). IFNs vs mass cytometry gated subpopulations beta regression. Results of beta regression analysis of relative frequency data for cell subpopulations defined by manual gating against plasma levels of IFNs in COVID-19-positive samples.

Supplementary Data 15 (separate file). Antibodies used in mass cytometry. List of antibodies used in mass cytometry. Column A indicates the antibody target, column B indicates the element conjugated to the antibody, column C indicates the mass of the element, column D indicates the manufacturer, column E indicates the catalog number, column F indicates the clone number, and column G indicates the type of stain protocol used (fixed, live or fixed with permeabilization).

Supplementary Data 16 (separate file). IFNs vs plasma metabolomics correlations. Results of Spearman correlation analysis between plasma levels of IFNs and plasma metabolomics data in COVID-19-positive samples.

Supplementary Data 17 (separate file). IFNs vs RBC metabolomics correlations. Results of Spearman correlation analysis between plasma levels of IFNs and red blood cell (RBC) metabolomics data in COVID-19-positive samples.

Supplementary Data 18 (separate file). IFNs vs clinical and demographics metadata. Results of regression analysis for plasma levels of IFNs by clinical demographic features.

Supplementary Data 19 (separate file). IFNs vs clinical laboratories. Results of Spearman correlation analysis between plasma levels of IFNs and clinical laboratory measurements in COVID-19-positive samples.

Supplementary Data 20 (separate file). IFNs vs MSD cytokines correlations. Results of Spearman correlation analysis between plasma levels of IFNs and immune factors measured using the MesoScale Discovery (MSD) platform in COVID-19-positive samples.

SI REFERENCES

1. Sullivan KD, *et al.* (2021) The COVIDome Explorer researcher portal. *Cell Rep*:109527.
2. Deutsch EW, *et al.* (2017) The ProteomeXchange consortium in 2017: supporting the cultural change in proteomics public data deposition. *Nucleic Acids Res* 45(D1):D1100-D1106.
3. Perez-Riverol Y, *et al.* (2019) The PRIDE database and related tools and resources in 2019: improving support for quantification data. *Nucleic Acids Res* 47(D1):D442-D450.
4. Spidlen J, Breuer K, Rosenberg C, Kotecha N, & Brinkman RR (2012) FlowRepository: a resource of annotated flow cytometry datasets associated with peer-reviewed publications. *Cytometry A* 81(9):727-731.
5. Gold L, Walker JJ, Wilcox SK, & Williams S (2012) Advances in human proteomics at high scale with the SOMAscan proteomics platform. *N Biotechnol* 29(5):543-549.
6. Johnson M, *et al.* (2020) Evaluation of a novel multiplexed assay for determining IgG levels and functional activity to SARS-CoV-2. *J Clin Virol* 130:104572.
7. Finck R, *et al.* (2013) Normalization of mass cytometry data with bead standards. *Cytometry A* 83(5):483-494.
8. Zunder ER, *et al.* (2015) Palladium-based mass tag cell barcoding with a doublet-filtering scheme and single-cell deconvolution algorithm. *Nature protocols* 10(2):316-333.
9. Schuyler RP, *et al.* (2019) Minimizing Batch Effects in Mass Cytometry Data. *Front Immunol* 10:2367.
10. Nemkov T, Reisz JA, Gehrke S, Hansen KC, & D'Alessandro A (2019) High-Throughput Metabolomics: Isocratic and Gradient Mass Spectrometry-Based Methods. *Methods in molecular biology* 1978:13-26.
11. Bushnell B, Rood J, & Singer E (2017) BBMerge - Accurate paired shotgun read merging via overlap. *PLoS One* 12(10):e0185056.
12. Kim D, Paggi JM, Park C, Bennett C, & Salzberg SL (2019) Graph-based genome alignment and genotyping with HISAT2 and HISAT-genotype. *Nature biotechnology* 37(8):907-915.

13. Li H, *et al.* (2009) The Sequence Alignment/Map format and SAMtools. *Bioinformatics* 25(16):2078-2079.
14. Anders S, Pyl PT, & Huber W (2015) HTSeq--a Python framework to work with high-throughput sequencing data. *Bioinformatics* 31(2):166-169.
15. Love MI, Huber W, & Anders S (2014) Moderated estimation of fold change and dispersion for RNA-seq data with DESeq2. *Genome biology* 15(12):550.
16. Guo K, *et al.* (2020) Qualitative Differences Between the IFNalpha subtypes and IFNbeta Influence Chronic Mucosal HIV-1 Pathogenesis. *PLoS Pathog* 16(10):e1008986.
17. Liao Y, Smyth GK, & Shi W (2014) featureCounts: an efficient general purpose program for assigning sequence reads to genomic features. *Bioinformatics* 30(7):923-930.
18. Robinson MD, McCarthy DJ, & Smyth GK (2010) edgeR: a Bioconductor package for differential expression analysis of digital gene expression data. *Bioinformatics* 26(1):139-140.
19. De Livera AM, *et al.* (2012) Normalizing and integrating metabolomics data. *Anal Chem* 84(24):10768-10776.
20. Chen H, *et al.* (2016) Cytofkit: A Bioconductor Package for an Integrated Mass Cytometry Data Analysis Pipeline. *PLoS Comput Biol* 12(9):e1005112.
21. Hahne F, *et al.* (2009) flowCore: a Bioconductor package for high throughput flow cytometry. *BMC bioinformatics* 10:106.
22. Krijthe J (2015) Rtsne: T-Distributed Stochastic Neighbor Embedding using a Barnes-Hut Implementation).
23. Levine JH, *et al.* (2015) Data-Driven Phenotypic Dissection of AML Reveals Progenitor-like Cells that Correlate with Prognosis. *Cell* 162(1):184-197.
24. Galbraith MD, *et al.* (2021) Seroconversion stages COVID19 into distinct pathophysiological states. *Elife* 10.
25. Valikangas T, Suomi T, & Elo LL (2018) A systematic evaluation of normalization methods in quantitative label-free proteomics. *Brief Bioinform* 19(1):1-11.
26. Chong J, Wishart DS, & Xia J (2019) Using MetaboAnalyst 4.0 for Comprehensive and Integrative Metabolomics Data Analysis. *Curr Protoc Bioinformatics* 68(1):e86.
27. Subramanian A, *et al.* (2005) Gene set enrichment analysis: a knowledge-based approach for interpreting genome-wide expression profiles. *Proc Natl Acad Sci U S A* 102(43):15545-15550.
28. Sergushichev AA (2016) An algorithm for fast preranked gene set enrichment analysis using cumulative statistic calculation. *bioRxiv*:060012.
29. Liberzon A, *et al.* (2015) The Molecular Signatures Database (MSigDB) hallmark gene set collection. *Cell Syst* 1(6):417-425.
30. Cribari-Neto F & Zeileis A (2010) Beta Regression in R. *Journal of statistical software* 34(2):24.
31. Wickham H (2016) *ggplot2: Elegant Graphics for Data Analysis* (Springer-Verlag New York).
32. Gu Z, Eils R, & Schlesner M (2016) Complex heatmaps reveal patterns and correlations in multidimensional genomic data. *Bioinformatics* 32(18):2847-2849.
33. Lüdecke D (2018) ggeffects: Tidy Data Frames of Marginal Effects from Regression Models. *The Journal of Open Source Software* 3(26):772.
34. Harrell FEH (2020) Hmisc: Harrell Miscellaneous.).
